# Chalcone Methoxy Derivatives Exhibit Antiproliferative and Proapoptotic Activity on Canine Lymphoma and Leukemia Cells

**DOI:** 10.3390/molecules25194362

**Published:** 2020-09-23

**Authors:** Aleksandra Pawlak, Marta Henklewska, Beatriz Hernández Suárez, Mateusz Łużny, Ewa Kozłowska, Bożena Obmińska-Mrukowicz, Tomasz Janeczko

**Affiliations:** 1 Department of Pharmacology and Toxicology, Wrocław University of Environmental and Life Sciences, C.K. Norwida 31, 50-375 Wrocław, Poland; marta.henklewska@upwr.edu.pl (M.H.); beatriz.hernandez-suarez@upwr.edu.pl (B.H.S.); b.mrukowicz@gmail.com (B.O.-M.); 2 Department of Chemistry, Wrocław University of Environmental and Life Sciences, Norwida 25, 50-375 Wrocław, Poland; mateusz.luzny@upwr.edu.pl (M.Ł.); ewa.kozlowska1@upwr.edu.pl (E.K.); tomasz.janeczko@upwr.edu.pl (T.J.)

**Keywords:** chalcones, apoptosis, DNA damage, anticancer activity, canine cancer cell lines

## Abstract

Chalcones are interesting candidates for anti-cancer drugs due to the ease of their synthesis and their extensive biological activity. The study presents antitumor activity of newly synthesized chalcone analogues with a methoxy group on a panel of canine lymphoma and leukemia cell lines. The antiproliferative effect of the 2′-hydroxychalcone and its methoxylated derivatives was evaluated in MTT assay after 48 h of treatment in different concentrations. The proapoptotic activity was studied by cytometric analysis of cells stained with Annexin V/FITC and propidium iodide and by measure caspases 3/7 and 8 activation. The DNA damage was evaluated by Western blot analysis of phosphorylated histone H2AX. The new compounds had selective antiproliferative activity against the studied cell lines, the most effective were the 2′-hydroxy-2″,5″-dimethoxychalcone and 2′-hydroxy-4′,6′-dimethoxychalcone. 2′-Hydroxychalcone and the two most active derivatives induced apoptosis and caspases participation, but some percentage of necrotic cells was also observed. Comparing phosphatidylserine externalization after treatment with the different compounds it was noted that the addition of two methoxy groups increased the proapoptotic potential. The most active compounds triggered DNA damage even in the cell lines resistant to chalcone-induced apoptosis. The results confirmed that the analogues could have anticancer potential in the treatment of canine lymphoma or leukemia.

## 1. Introduction

The search for novel natural or synthetic compounds with potential antitumor activity is one of the major goals of contemporary oncology. The research involves both known compounds with proven biological activity as well as newly synthesized molecules that are structural analogues of biologically active substances already used in the treatment of cancer. Interest is aroused by compounds that may constitute the basis of chemotherapy in the future, as well as compounds that may support therapy or sensitize cancer cells to classic cytostatic drugs. Because the high toxicity of many anticancer substances (particularly bone marrow toxicity) significantly reduces the effectiveness of the treatment, to maximize the efficacy and reduce adverse effects, chemotherapy is usually based on a combination of drugs with various molecular mechanisms of action and different side effects. With regard to this principle, research focusing on the antitumor activity of new natural compounds or their derivatives is highly justified. Such a potentially interesting group of compounds may be chalcones, an essential group of natural compounds classified as flavonoids [1]. Their typical structural feature is an open, α,β-unsaturated three-carbon fragment connecting two aromatic rings [2]. They are yellow [3] and commonly occur in the world of plants: in citrus fruits, vegetables and spices [4,5]. Chalcones (1-(2′-hydroxyphenyl)-3-phenylprop-2-en-1-ones) and their derivatives are often obtained from natural sources or as a result of chemical synthesis [6,7]. The most commonly used chemical synthesis method is the Claisen–Schmidt condensation of the corresponding aromatic aldehyde and 2′-hydroxyacetophenones in alkaline or acid catalysis [8,9,10].

The great interest in chalcones is influenced by the ease of synthesis of these compounds and the extensive biological activity characteristics they exhibit: in particular antiviral, anthelmintic, antibacterial, antiprotozoal, insecticidal, antiulcer, cytotoxic, and anticancer [11,12,13]. Due to the confirmed therapeutic effect of chalcones on human cancer, we decided to check their activity against canine cancer cells.

Cancer in dogs is a major challenge in modern veterinary medicine and research into the search for new cancer therapies is highly needed [14]. On the other hand, of the various animal species that develop cancer (e.g., cats, horses, rabbits, ferrets), dogs turned out to be the most appropriate model for comparative research due to their body size, life expectancy, cancer incidence and sharing of living conditions with humans. Moreover, dogs together with mice and rats, are popular laboratory animals necessary for different types of toxicological studies [15].

In conclusion, the presented study examined the antitumor activity of new chalcone analogues with the methoxy group on selected canine lymphoma and leukemia cell lines. After the hydroxyl group, the methoxy group is the second most commonly identified group in natural flavonoid compounds, and because of this, we decided to test a series of chalcones containing a different amount and places of methoxy group substitution. The cytotoxic, antiproliferative and proapoptotic effects of newly synthesized compounds were analyzed. The study showed that the obtained analogues could have anticancer potential in the treatment of canine lymphoma or leukemia.

## 2. Results

### 2.1. Chalcone Analogues with the Methoxy Group Have Selective Antiproliferative Activity on Canine Cancer Cell Lines

**Figure 1:**
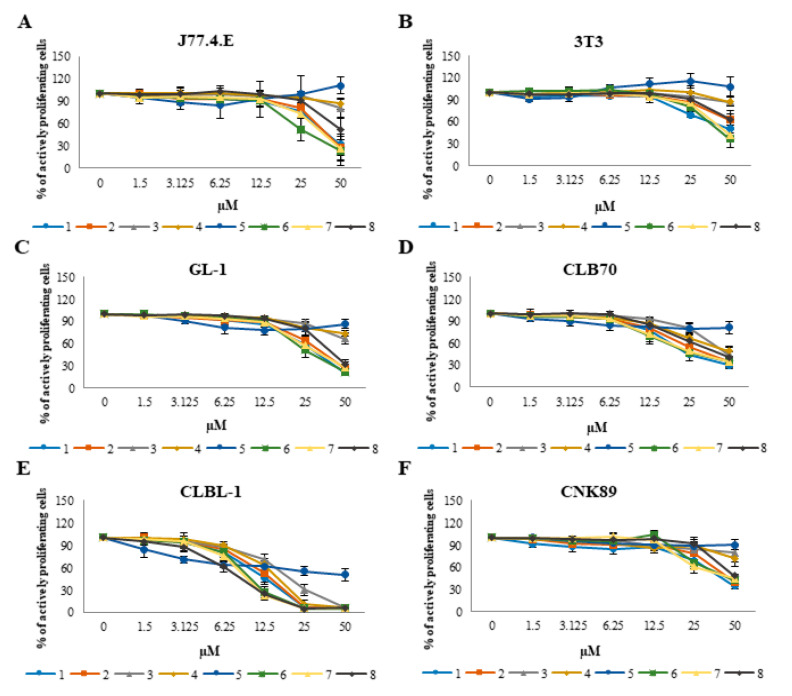
Concentration-dependent curves presenting the effects of the tested chalcones on viability of normal (**A**,**B**) and canine cancer cell lines (**C**–**F**) after 48 h of incubation with different concentrations of the individual chalcones (1.5, 3.125, 6.25, 12.5, 25 and 50 µM). The values are means from three independent experiments.

First, an assay was performed to determine whether the tested chalcones have antiproliferative activity against normal cells, and if the position of the substituents in the structure has an influence on the potency of such effects. The study showed that normal cells, represented by the NIH/3T3 and J774E.1 cell line, were less sensitive to the antiproliferative action of the tested chalcones than cancer cells. The IC50 value (concentration that inhibits the proliferation of 50% of cells) was determined only for two compounds in case of the NIH/3T3 cell line and four compounds in case of the J77.4.E cell line. These four compounds (**1**, **2**, **6** and **7**) proved to be the most active also in research with the use of cancer cell lines. The curves showing the inhibition of normal cell growth by individual compounds are shown in Figure 1A,B.

While the tested compounds did not show a significant inhibitory effect on the growth of normal cells, their potency in relation to cancer cell lines was visibly different. All the tested compounds had a particularly strong effect on the CLBL-1 cell line, representing the most common type of lymphoma in dogs—diffuse large B-cell lymphoma (DLBCL). Other B lymphocytes, from chronic lymphocytic leukemia (CLB70) were also more sensitive than the others. Natural killer (NK) cells obtained from the rare and refractory form of canine lymphoma were found to be the most resistant. Similarly, but slightly more strongly, the tested compounds acted on the GL-1 cell line, representing canine acute leukemia.

Detailed results in the form of a table containing IC50 values for all tested compounds on all tested cell lines are presented in Table 1. Growth inhibition curves of canine cancer cell lines are shown in Figure 1C–F.

### 2.2. Chalcone Analogues with the Methoxy Group, at Least Partially, Kill Cancer Cells through Apoptosis

**Figure 2:**
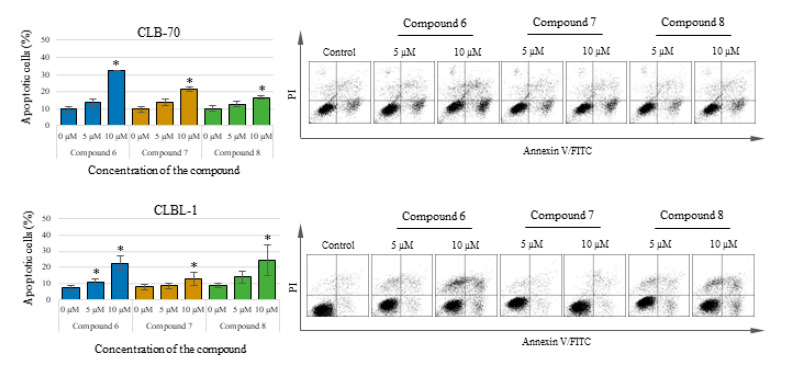
Percentage of annexin V positive (apoptotic) cells after 48 h of incubation with the culture medium alone (0) or 5 and 10 µM of the tested chalcones. On the right: representative dot-plots of annexin V/PI staining. The values are means of four independent experiments. * Considered significant in comparison to the control (*p* < 0.05).

The substances with the strongest activity in the MTT test were selected for further research on the nature of cell death induced by the studied compounds. To determine the type of cell death induced by the tested chalcones, in the first place, the assay for detection of early signs of apoptosis, which is the externalization of phosphatidylserine, was performed. The study used the two cell lines which were the most sensitive to the tested compounds: CLBL-1 and CLB70 and the compounds with the strongest action: 2′-hydroxy-2″,5″-dimethoxychalcone (compound **6**), 2′-hydroxy-4′,6′-dimethoxychalcone (compound **7**) and initial compound (compound **8**). It was shown that cell death takes place by apoptosis, as confirmed by the statistical analysis of the results. After 48 h of incubation with the tested chalcone analogues at relatively low concentrations of 5 and 10 µM (concentrations harmless to normal cells), both the CLBL-1 and CLB70 cells were found to have a positive reaction with Annexin V, which is evidence of phosphatidylserine externalization and thus the ongoing process of apoptosis. As a result of the actions of the tested compounds, in addition to the population of apoptotic cells, the presence of cells that have already lost the integrity of the cell membrane was found (such a population of cells can also be referred as necrotic). The presence of cells staining positively with propidium iodide, a compound permanently staining the DNA of dead cells, can be explained by the relatively long incubation time used in the study (some of the cells that died by apoptosis had already lost their integrity too). Another explanation assumes that apoptosis is only one of the processes that cells undergo under the influence of the tested chalcones. Detailed results and representative dot plots are shown in Figure 2.

**Figure 3:**
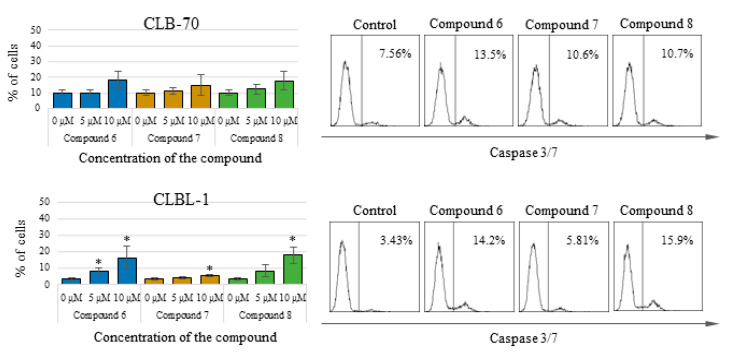
Percentage of cells with active caspase 3/7 after 48 h of incubation with the culture medium alone (0) or 5 and 10 µM of the tested chalcones. On the right: representative histograms for caspase 3/7 activation. The values are means of four independent experiments. * considered significant in comparison to the control (*p* < 0.05).

**Figure 4:**
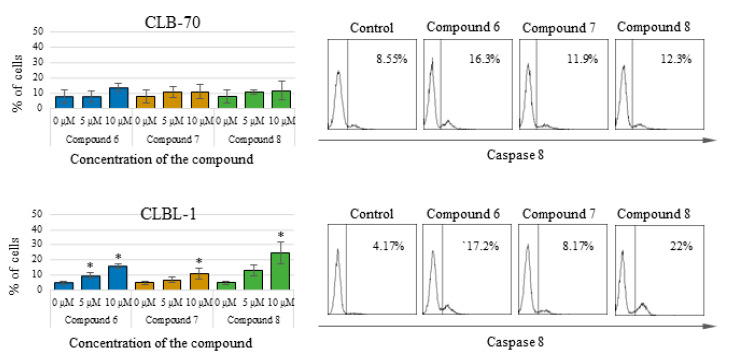
Percentage of cells with active caspase 8 after 48 h of incubation with the culture medium alone (0) or 5 and 10 µM of the tested chalcones. On the right: representative histograms for caspase 8 activation. The values are means of four independent experiments. * considered significant in comparison to the control (*p* < 0.05).

To better explain the type of cell death that cancer cells undergo after treatment with the tested compounds, specific assays were carried out to determine the activity of caspases—the most important enzymes involved in the process of apoptosis. By flow cytometry, it was found that in CLBL-1 and CLB70 cells, after treatment with the tested chalcones, activation of the effector caspase 3 and initiator caspase 8 occur. The observed activation of caspase 3, the most important caspase in the caspase-dependent pathway of apoptosis was not massive and covered only a small percent of cells (about 15%). This result confirms the induction of apoptosis in the cells, but at the same time, indicates that apoptosis is not the only type of cell death of cells treated with the tested compounds. This result is consistent with the result of the Annexin V staining, in which similar percentage of necrotic cells was observed. 2′-Hydroxy-2″,5″-dimethoxychalcone (**6**) and initial compound activated caspase 3 the most strongly, whereas 2′-hydroxy-4′,6′-dimethoxychalcone (**7**) had a slightly weaker effect. More pronounced activation of caspase 3 occurred in the CLBL-1 cell line. Similar results were obtained for caspase 8, which was more strongly activated in the CLBL-1 cells, showing a statistically significantly higher level after treatment with all three compounds. Detailed results along with representative histograms are presented in Figure 3 (caspase 3 activation) and Figure 4 (caspase 8 activation).

### 2.3. Tested Chalcone Analogues Trigger DNA Damage in Canine Lymphoma/Leukemia Cell Lines

**Figure 5:**
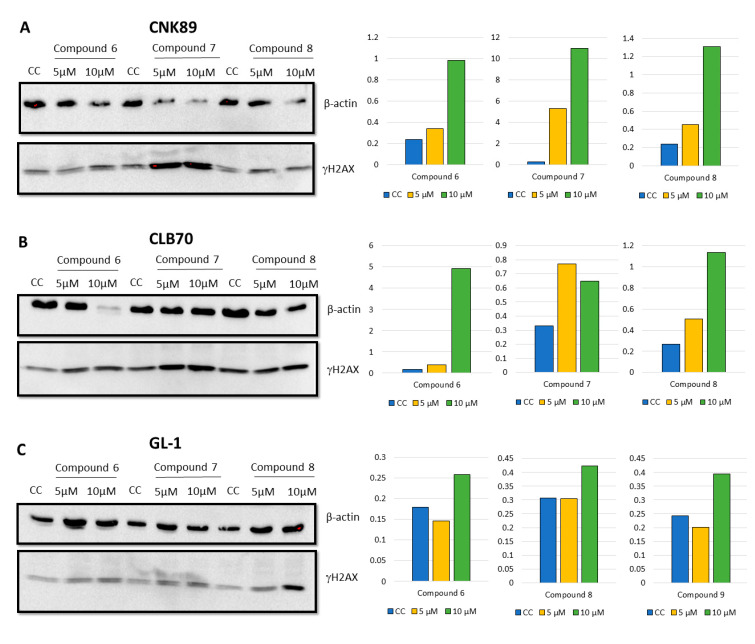
Western blot analysis for phosphorylated H2AX of CNK89, CLB70 and GL-1 cell lines after 48 h of incubation with different concentrations (5 and 10 μM) of compound **6**–**8** (**A**–**C**).

Because the degree of apoptosis induction detected in Annexin V binding assay was lower than expected and did not correlate perfectly with the results of cytotoxicity tests, we decided to check the level of DNA damage as additional marker of potentially lethal events in the cells (leading to cell death and the initiation of the apoptosis process). The study showed that the incubation of CLB70, CNK89 and even the resistant GL-1 cell lines with 2′-hydroxy-2″,5″-dimethoxychalcone (**6**), 2′-hydroxy-4′,6′-dimethoxychalcone (**7**) and initial 2′-hydroxychalcone (**8**) provoked the DNA damaged manifested by increased histone H2AX phosphorylation. Detailed results are presented in Figure 5.

## 3. Discussion

The aim of the presented study was to obtain chalcone analogues with better anticancer properties compared to the parent compound. Because a significant problem in the use of various flavonoids is their poor availability, it was decided to focus on improving properties such as the ability to enter the cell. Therefore, the modification that was made was to create analogues containing a methoxy group instead of a hydroxyl group. Such a change can have a dual effect. Often, the conversion of a hydroxyl to a methoxy group results in a decrease in the biological activity of the flavonoid compound in vitro [16,17]. On the other hand, it was proven that methoxylated flavonoids have the ability to easily penetrate the cells [18]. For example, it was demonstrated that after transferring lung cell lines to the medium containing dimethoxyflavones, within five minutes, the cells accumulated 30–50 times more test compounds than were present in the surrounding buffer [19]. The intracellular transport of 5,7-dimethoxyflavone was approximately 10-fold higher than that of chrysin (5,7-dihydroxyflavone). Moreover, chrysin was rapidly metabolized by the human liver (S9 fraction), with no parent compound remaining after a 20-min incubation. In contrast, 5,7-dimethoxyflavone was metabolically stable over the whole 60-min time-course studied [20,21]. Better absorption and distribution, as well as the prolonged metabolism of methoxylated flavonoids, makes them even more promising compounds. Furthermore, their oral bioavailability exceeds the bioavailability of hydroxylated flavonoids, which makes them easy to administer [22]. Based on the knowledge of this phenomenon, we decided to create derivatives with a methoxy group and see how such modification affects the antitumor activity in vitro.

In our research, we first decided to check whether the obtained analogues show selective antiproliferative activity against cancer cells. As shown in Figure 2, it turned out that in fact, canine leukemia/lymphoma cells are more sensitive to the action of the obtained chalcones than the normal cell lines used in the study. The potency of the antiproliferative activity of the tested compounds was higher in relation to the cancer cell lines, but the same compounds that most strongly inhibited the proliferation of cancer cells were also the most toxic to normal cells. These compounds were: 2′-hydroxy-2″-methoxychalcone (**1**), 2′-hydroxy-3″-methoxychalcone (**2**), 2′-hydroxy-2″,5″-dimethoxychalcone (**6**) and 2′-hydroxy-4′,6′-dimethoxychalcone (**7**), the most active of which were the last two. Analyzing the differences in the chemical structure of individual compounds, at this stage of research, it can be concluded that the presence of two methoxy groups, regardless of their position, is responsible for the strength of the cytotoxic activity on normal cell lines. Regarding cancer cell lines, the differences in the potency of all four compounds mentioned above are too small to find conclusions. It seems, however, that the strongest antitumor activity is shown by compounds containing one methoxy group, but only in the position 2 or 3 of the B ring. Derivatives containing a methoxy group in 4 position or containing 3 or more such groups – are characterized by weaker antiproliferative activity. Looking at the table with IC50 values, one more interesting fact can be seen—the lowest IC50 value was observed in relation to the CLBL-1 cell line, for the parent compound—2′-hydroxychalcone (**8**). This effect was observed only for the CLBL-1 cell line and this value does not differ statistically from the values for 2′-hydroxy-2″,5″-dimethoxychalcone (**6**) and 2′-hydroxy-4′,6′-dimethoxychalcone (**7**). The explanation for this observation may be the fact that the CLBL-1 cell line was characterized by very strong sensitivity to all the tested compounds, therefore it is impossible in this case to capture the influence of small differences in the structure of the compounds on their activity against this cell line. However, the observed high sensitivity of the CLBL-1 cell line to the flavonoids may be a valuable therapeutic indication. This cell line was obtained from a dog with the most common type of lymphoma, and if it reflects the sensitivity of the cells of this type of disease then it may be possible to consider more thorough studies on the potential use of this group of compounds in therapy in dogs.

The proapoptotic effect of flavonoids or specifically chalcones is still a frequently discussed subject of scientific research. Numerous scientific reports describe the different strengths and mechanisms of proapoptotic action of compounds from this group [23,24,25,26]. In our research, we focused not on studying the mechanism of the induced apoptosis, but on comparing the strength with which various methoxy derivatives of chalcones induce suicidal cell death. Comparing proapoptotic activity 2′-hydroxy-2″,5″-dimethoxychalcone (**6**), 2′-hydroxy-4′,6′-dimethoxychalcone (**7**) and initial 2′-hydroxychalcone (**8**) it was noted that the presence of methoxy groups in positions 2 and 5 in the B ring, affects the potency of the proapoptotic action of the obtained derivatives. 2′-Hydroxy-2″,5″-dimethoxychalcone (**6**) showed a clearly stronger proapoptotic activity than the parent compound both in the annexin V and in caspase 3/7 and 8 activity tests. In the case of the CLBL-1 cell line, this difference was less visible, and in relation to caspase 8 the parent compound more strongly caused its cleavage. The presence of two methoxy groups, but at positions 4′ and 6′ (compound **7**) also increased the proapoptotic activity compared to the parent compound, as clearly seen in studies using the CLB70 cell line. However, this difference could not be confirmed by test using the CLBL-1 cell line. Another important observation was that the potency of the cytotoxic activity of the obtained derivatives with the methoxy group observed in the MTT test significantly exceeded that observed in the apoptosis tests. Because the MTT assay assesses the antiproliferative activity of the tested compounds, it can be concluded that the obtained chalcone derivatives strongly inhibited the proliferation of canine lymphoma/leukemia cells and the induction of apoptosis itself was slightly weaker. Such action of various chalcones has already been described. In addition to apoptosis, the compounds belonging to the chalcones have also been found to cause cell cycle arrest, which may be both the cause and the effect of the proapoptotic action of this group of compounds [27,28,29,30]. Because the mechanism of antitumor action of chalcones, apart from inhibiting cell proliferation and induction of apoptosis, also includes regulation of cell survival, invasiveness or angiogenesis [31,32], we decided to additionally check how the introduction of the methoxy group impact DNA damage. One of the mechanisms identified for this action is related to the chalcones’ ability to selectively target of the ubiquitin-proteasome system (UPS) [31,32]. Published evidence suggests that the presence of an α,β-unsaturated carbonyl group is the key molecular determinant conferring UPS- and DUBs (deubiquitinating enzymes)-inhibitory activity of different chalcones [33]. Because DUBs are crucial in regulating a variety of cellular pathways, including cell growth and proliferation, apoptosis, protein quality control, DNA repair and transcription [34], the ability to inhibit them is an important feature of a potential anti-cancer compound. Inhibiting DUBs activity impairs 20S proteasome proteolytic activities and the cellular deubiquitinating enzymes, leading to increased accumulation of ubiquitinated proteins and consequently causes endoplasmic reticulum stress and cell death [35,36]. The effect may be also DNA damage. In the present study it has been clearly demonstrated that both the parent compound and the two strongest derivatives cause DNA damage observed as an increase in histone H2AX phosphorylation. Despite the use in this assay of cell lines (CNK89 and GL-1) slightly less sensitive to the action of chalcones it cannot be unequivocally stated that the obtained derivatives acted more strongly than the parent compound. Such observations can be explained by the lack of influence of methoxy groups on the formation of DNA damage (the presence of an α,β-unsaturated carbonyl group is rather responsible for such action) and the fact that the observed DNA damage was to some extent an effect of the ongoing apoptosis. However, the strength and extent of the DNA damage found, even in the cancer cell lines more resistant to chalcones, indicates that at least in part, the DNA damage was the cause of the death of the canine lymphoma/leukemia cells.

## 4. Materials and Methods

### 4.1. Compounds

**Figure 6:**
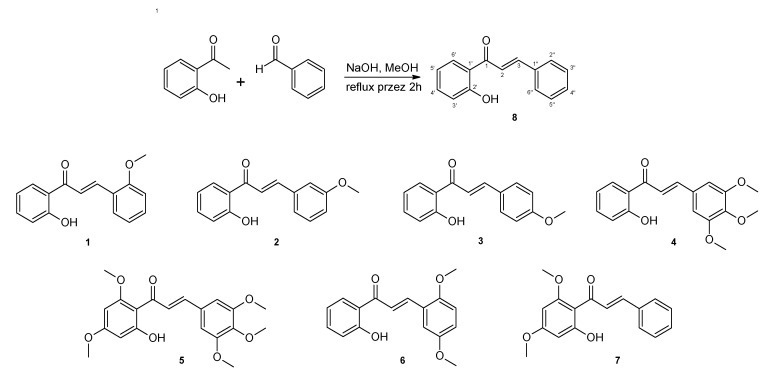
General scheme for the synthesis of chalcones and structures of obtained methoxy derivatives. The numbering of carbon atoms has been placed on compound **8**.

The chalcone analogues used in the study are numbered from 1 to 8. All were obtained via a Claisen–Schmidt condensation of an appropriate 2-hydroxyacetophenone and a substituted benzaldehyde dissolved in methanol in an alkaline environment at high temperature according to the procedure described previously [37,38,39]. The structures of the tested chalcones are shown in Figure 6.

The resulting compounds were characterized by the following NMR spectral data (Supplementary Materials Figures S1–S29):

*2′-Hydroxy-2″-methoxychalcone* (**1**). ^1^H NMR (600 MHz) (CDCl_3_) δ (ppm): 3.94 (s 3H, -OC*H*_3_), 6.94 (td, 1H, *J* = 7.8, 0.6 Hz, H-5′), 6.96 (d, 1H, *J* = 8.8 Hz, H-3″), 7.01 (t, 1H, *J* = 7.5 Hz, H-5″), 7.04 (dd, 1H, *J* = 8.3, 0.6 Hz, H-3′), 7.41 (ddd, 1H, *J* = 8.6, 7.9, 1.5 Hz, H-4″), 7.49 (t, 1H, *J* =8.8, 7.8, 1.4 Hz, H-4′); 7.65 (dd, 1H, *J* = 7.6, 1.3 Hz, H-6″), 7.78 (d, 1H, *J* = 15.6 Hz, H-2), 7.93 (dd, 1H, *J* = 8.0, 1.3 Hz, H-6′), 8.23 (d, 1H, *J* = 15.6 Hz, H-3); 12.95 (s, 1H, -O*H*). ^13^C NMR (151 MHz, CDCl_3_) δ = 55.73 (-O*C*H_3_), 111.45 (C-3″), 118.67 (C-3′), 118.88 (C-5′), 120.33 (C-1′), 120.94 (C-2), 120.94 (C-5″), 123.76 (C-1″), 129.77 (C-6′), 129.84 (C-6″), 132.34 (C-4″), 136.28 (C-4′), 141.27 (C-3), 159.17 (C-2″), 163.71 (C-2′), 194.44 (C-1).

*2′-Hydroxy-3″-methoxychalcone* (**2**). ^1^H NMR (600 MHz) (CDCl_3_) δ (ppm): 3.87 (s, 3H, -OC*H*_3_), 6.95 (ddd, 1H, *J* = 8.1, 7.1, 1.1 Hz, H-5′), 6.99 (dd, 1H, *J* = 8.2, 2.4 Hz, H-4″), 7.04 (dd, 1H, *J* = 8.4, 0.9 Hz, H-3′), 7.17 (dd, 1H, *J* = 2.2, 1.1 Hz, H-2″), 7.27 (d, 1H, *J* = 7.7 Hz, H-6″), 7.36 (t, 1H, *J* = 7.9 Hz, H-5″), 7.51 (ddd, 1H, *J* = 8.5, 7.2, 1.3 Hz, 1H, H-4′), 7.64 (d, 1H, *J* = 15.5 Hz, H-2), 7.89 (d, 1H, *J* = 15.5 Hz, H-3), 7.92 (dd, 1H, *J* = 8.1, 1.4 Hz, H-6′), 12.80 (s, 1H, -O*H*). ^13^C NMR (151 MHz, CDCl_3_) δ = 55.55 (-O*C*H_3_), 113.87 (C-2″), 116.76 (C-4″), 118.80 (C-3′), 119.01 (C-5′), 120.16 (C-1′), 120.58 (C-2), 121.43 (C-6″), 129.81 (C-6′), 130.19 (C-5″), 136.12 (C-1″), 136.58 (C-4′) 145.53 (C-3), 160.14 (C-3″), 163.75 (C-2′), 193.86 (C-1).

*2′-Hydroxy-4″-methoxychalcone* (**3**). ^1^H NMR (600 MHz) (CDCl_3_) δ (ppm): 3.87 (s, 3H, -OC*H*_3_), 6.94 (ddd, 1H, *J* = 8.1, 7.1, 1.0 Hz, H-5′), 6.94–6.97 (m, 2H, H-3″, H-5″), 7.02 (dd, 1H, *J* = 8.3, 0.8 Hz, H-3′), 7.49 (ddd, 1H, *J* = 8.5, 7.1, 1.5 Hz, H-4′), 7.54 (d, 1H, *J* = 15.4 Hz, H-2), 7.63 (m, 2H, H-2″, H-6″), 7.90 (d, 1H, *J* = 15.0 Hz, H-3), 7.93 (dd, 1H, *J* = 8.0, 1.3 Hz, H-6′), 12.95 (s, 1H, -O*H*). ^13^C NMR (151 MHz, CDCl_3_) δ = 55.59 (-O*C*H_3_), 114.66 (C-3″,-5″), 117.74 (C-2), 118.72 (C-3′), 118.89 (C-5′), 120.26 (C-1′), 127.49 (C-1″), 129.67 (C-6′), 130.69 (C-2″, C-6″), 136.28 (C-4′), 145.50 (C-3), 162.17 (C-4″), 163.69 (C-2′), 193.82 (C-1).

*2′-Hydroxy-3″,4″,5″-trimethoxychalcone* (**4**). ^1^H NMR (600 MHz) (CDCl_3_) δ (ppm): 3.91 (s, 3H, C-4″-OC*H*_3_), 3.94 (s, 6H, C-3″-OC*H*_3_ and C-5″-OC*H*_3_), 6.89 (s, 2H, H-2″ and H-6″), 6.96 (ddd, 1H, *J* = 8.0, 7.2, 1.0 Hz, H-5′), 7.04 (dd, 1H, *J* = 8.4, 1.0 Hz, H-3′), 7.51 (ddd, 1H, *J* = 8.3, 7.1, 1.4 Hz, H-4′), 7.54 (d, 1H, *J* = 15.4 Hz, H-2), 7.85 (d, 1H, *J* = 15.5 Hz, H-3), 7.93 (dd, 1H, *J* = 8.1, 1.5 Hz, H-6′), 12.84 (s, 1H, -O*H*). ^13^C NMR (151 MHz, CDCl_3_) δ = 56.42 (C-2″-O*C*H_3_ and C-6″-O*C*H_3_), 61.19 (C-4″-O*C*H_3_), 106.08 (C-2″, C-6″), 118.81 (C-3′), 118.95 (C-5′), 119.41 (C-2), 120.16 (C-1′), 129.74 (C-6′), 130.20 (C-1″), 136.52 (C-4′), 140.97 (C-4″), 145.79 (C-3), 153.68 (C-3″, C-5″), 163.73 (C-2′), 193.67 (C-1).

*2′-Hydroxy-4′,6′,3″,4″,5″-pentamethoxychalcone* (**5**). ^1^H NMR (600 MHz) (CDCl_3_) δ (ppm): 3.84 (s, 3H, C-4′-OC*H*_3_), 3.90 (s, 3H, C-4″-OC*H*_3_), 3.91 (s, 3H, C-6′-OC*H*_3_), 3.91 (s, 6H, C-2″-OC*H*_3_ and C-6″-OC*H*_3_), 5.96 (d, 1H, *J* = 2.3 Hz, H-5′), 6.11 (d, 1H, *J* = 2.3 Hz, H-3′), 6.84 (s, 2H, H-2″, H-6″), 7.70 (d, 1H, *J* = 15.5 Hz, H-3), 7.80 (d, 1H, *J* = 15.5 Hz, H-2), 14.31 (s, 1H, -O*H*). ^13^C NMR (151 MHz, CDCl_3_) δ = 55.75 (C-4′-O*C*H_3_), 55.94 (C-6′-O*C*H_3_), 56.27 (C-2″-O*C*H_3_ and C-6″-O*C*H_3_), 61.15 (C-4″-O*C*H_3_), 91.46 (C-5′), 93.97 (C-3′), 105.69 (C-2″, C-6″), 106.45 (C-1′), 127.06 (C-2), 131.28 (C-1″), 140.21 (C-4″), 142.54 (C-3), 153.54 (C-3″, C-5″), 162.53 (C-6′), 166.34 (C-4′), 169.56 (C-2′), 192.49 (C-1).

*2′-Hydroxy-2″,5″-dimethoxychalcone* (**6**). ^1^H NMR (600 MHz) (CDCl_3_) δ (ppm): 3.83 (s, 3H, C-5″-OC*H*_3_), 3.90 (s, 3H, C-2″-OC*H*_3_), 6.90 (d, 1H, *J* = 9.0 Hz, H-3″), 6.94 (ddd, 1H, *J* = 9.0, 7.2, 0.9 Hz, H-5′), 6.97 (dd, 1H, *J* = 9.0, 3.1 Hz, H-4″), 7.02 (dd, 1H, *J* = 8.4, 1.0 Hz, H-3′), 7.17 (d, 1H, *J* = 3.1 Hz, H-6″), 7.49 (ddd, 1H, *J* = 8.3, 7.1, 1.5 Hz, H-4′), 7.75 (d, 1H, *J* = 15.6 Hz, H-2), 7.92 (dd, 1H, *J* = 8.1, 1.6 Hz, H-6′), 8.19 (d, 1H, *J* = 15.6 Hz, H-3), 12.92 (s, 1H, -O*H*). ^13^C NMR (151 MHz, CDCl_3_) δ = 56.02 (C-5″-O*C*H_3_), 56.27 (C-2″-O*C*H_3_), 112.63 (C-3″), 114.32 (C-6″), 117.80 (C-4″), 118.70 (C-3′), 118.91 (C-5′), 120.31 (C-1′), 121.17 (C-2), 124.31 (C-1″), 129.85 (C-6′), 136.34 (C-4′), 141.05 (C-3), 153.65 (C-5″), 153.73 (C-2), 163.71 (C-2′), 194.35 (C-1).

*2′-Hydroxy-4′,6′-dimethoxychalcone* (**7**). ^1^H NMR (600 MHz) (CDCl_3_) δ (ppm): 3.83 (s, 3H, C-4′-OC*H*_3_), 3.92 (s, 3H, C-6′-OC*H*_3_), 5.97 (d, 1H, *J* = 2.4 Hz, H-5′), 6.11 (d, 1H, *J* = 2.4 Hz, H-3′), 7.36–7.43 (m, 3H, H-3″, H-4″, H-5″), 7.58–7.62 (m, 2H, H-2″, H-6″), 7.79 (d, 1H, *J* = 15.6 Hz, H-3), 7.91 (d, 1H, *J* = 15.6 Hz, H-2), 14.29 (s, 1H, -O*H*). ^13^C NMR (151 MHz, CDCl_3_) δ = 55.72 (C-4″-O*C*H_3_), 55.99 (C-6′-O*C*H_3_), 91.41 (C-5′), 93.93 (C-3′), 106.47 (C-1′), 127.66 (C-2), 128.48 (C-2″ and C-6″), 129.00 (C-3″ and C-5″), 130.18 (C-4″), 135.70 (C-1″), 142.45 (C-3), 162.64 (C-6′), 166.37 (C-4′), 168.53 (C-2′), 192.77 (C-1).

*2′-Hydroxychalcone* (**8**). ^1^H NMR (600 MHz) (CDCl_3_) δ (ppm): 3.94 (s 3H, -OC*H*_3_), 6.94 (td, 1H, *J* = 7.8, 0.6 Hz, H-5′), 6.96 (d, 1H, *J* = 8.8 Hz, H-3″), 7.01 (t, 1H, *J* = 7.5 Hz, H-5″), 7.04 (dd, 1H, *J* = 8.3, 0.6 Hz, H-3′), 7.41 (ddd, 1H, *J* = 8.6, 7.9, 1.5 Hz, H-4″), 7.49 (t, 1H, *J* =8.8, 7.8, 1.4 Hz, H-4′); 7.65 (dd, 1H, *J* = 7.6, 1.3 Hz, H-6″), 7.78 (d, 1H, *J* = 15.6 Hz, H-2), 7.93 (dd, 1H, *J* = 8.0, 1.3 Hz, H-6′), 8.23 (d, 1H, *J* = 15.6 Hz, H-3); 12.95 (s, 1H, -O*H*). ^13^C NMR (151 MHz, CDCl_3_) δ = 55.73 (-O*C*H_3_), 111.45 (C-3″), 118.67 (C-3′), 118.88 (C-5′), 120.33 (C-1′), 120.94 (C-2), 120.94 (C-5″), 123.76 (C-1″), 129.77 (C-6′), 129.84 (C-6″), 132.34 (C-4″), 136.28 (C-4′), 141.27 (C-3), 159.17 (C-2″), 163.71 (C-2′), 194.44 (C-1).

### 4.2. Cell Lines and Cell Culture

The study involved the following normal, non-cancerous NIH/3T3 (fibroblasts) and J774E.1 (macrophages) murine cell lines and a panel of canine cancer cell lines: CLBL-1 (B-cell lymphoma), GL-1 (B/T-cell leukemia), CLB70 (B-cell chronic lymphocytic leukemia) and CNK89 (NK-cell lymphoma). The NIH/3T3 and J774E.1 cell lines were bought from the American Type Culture Collection (ATCC, Rockville, MD, USA). CLBL-1 was obtained from Barbara C. Ruetgen from the Institute of Immunology, Department of Pathobiology, University of Veterinary Medicine, Vienna, Austria [40], GL-1 was obtained from Yasuhito Fujino and Hajime Tsujimoto from the University of Tokyo, Department of Veterinary Internal Medicine [41,42] while CLB70 [43] and CNK89 (Grudzień et al., in press) were established in our laboratory.

The cell lines were maintained in RPMI 1640 (J774E.1, NIH/3T3, CLBL-1 and GL-1) (Institute of Immunology and Experimental Therapy, Polish Academy of Sciences, Wrocław, Poland) or Advanced RPMI (Gibco, Grand Island, NY, USA) (CLB70 and CNK89) culture medium supplemented with 2 mM L-glutamine (Sigma Aldrich, Steinheim, Germany), 100 U/mL penicillin, 100 μg/mL streptomycin (Sigma Aldrich, Steinheim, Germany), and 10–20% heat-inactivated fetal bovine serum—FBS (Gibco, Grand Island, NY, USA).

### 4.3. Cell Proliferation Assay

The cell proliferation was determined using the MTT test (Sigma Aldrich, Steinheim, Germany). In brief, 1 × 10^4^ (NIH/3T3 and J77.4.E) or 1 × 10^5^ (canine cancer cell lines) cells per well were seeded in a 96-well-plate (Thermo Fisher Scientific, Denmark), and the tested chalcones were added in the increasing concentrations (1.5, 3.125, 6.25, 12.5, 25 and 50 µM). After incubation for 48 h, 20 µL of MTT solution (5 mg/mL) were added to each well. After the contents dissolved, the optical density of wells was measured with a microplate reader (Spark, Tecan) at a reference wavelength of 570 nm. The values were means from three independent experiments (three wells each).

### 4.4. Western Blotting

For Western blot analysis the cells were seeded in a total of 5 × 10^6^ cells per 25 cm^2^ cell culture flasks and two selected concentration of the compounds were added. After 48 h incubation the cells were harvested, rinsed with cold PBS, suspended in a lysis buffer (50 mM Tris–HCl pH 7.5, 100 mM NaCl, 1% NP-40 and protease inhibitors set) and incubated for 20 min on ice. The suspensions were centrifuged at 10,000 rpm at 4 ˚C for 12 min. Then, a sodium dodecyl sulfate (SDS) sample buffer was added to clear supernatants, and the samples were boiled at 95 ˚C for 5 min and subjected to SDS-PAGE on 10–15% gel. For the tests, the resolved proteins were transferred to a PVDF membrane (Millipore, Billerica, MA, USA), using Semidry Transfer Cell (Bio-Rad, Hercules, CA, USA). After the transfer, the membrane was blocked overnight with 1% casein in TBS at 4 ˚C, and then incubated with primary antibody (dilution 1:2000) (Santa Cruz Biotechnology, USA) at room temperature for 1 h, followed by secondary horseradish peroxidase-labelled antibody (Dako, Denmark). The bound antibodies were visualized using ChemiDoc Touch Instruments (BioRad, Hercules, CA, USA). The anti-γH2A.X (ab26350) antibody was from Abcam (Cambridge, UK) while the anti-β actin (C-4) antibody was from Santa Cruz Biotechnology (Santa Cruz, CA, USA).

### 4.5. Apoptosis Assays by Flow Cytometry

After seeding at the same density as for cytotoxicity tests in 96-well plates (TPP, Trasadingen, Switzerland) the cells were incubated for 48 h with two selected concentrations of the tested chalcones (5 and 10 µM). For the phosphatidylserine externalization and membrane integrity test, cells were collected, suspended in a binding buffer and stained with Annexin V-FITC and PI (final PI concentration 1 µg/mL). To evaluate caspases 3/7 and 8 the cells were harvested, washed twice with PBS and stained according to the manufacturer’s instructions. Briefly, for active caspase 3/7 detection CellEventCaspase-3/7 Green Detection Reagent was added to the samples. For the detection of active caspase 8, FITC-IETD-fmk was added to the samples and the cells were incubated for 0.5 h. Then, the cells were washed twice and re-suspended in wash buffer. Flow cytometric analysis was immediately performed using a flow cytometer (FACS Calibur; Becton Dickinson, Biosciences, San Jose, CA, USA). CellQuest 3.lf. Software (Becton Dickinson, San Jose, CA, USA) was used for data analysis.

### 4.6. Statistical Analysis

All data are shown as means with standard deviations (SD). Statistical differences were analyzed using one-way ANOVA followed by Tukey’s multiple comparison test. Statistical analysis was performed with STATISTICA version 13.3 software (TIBCO Software Inc., Palo Alto, CA, USA). The results were considered significant at *p* < 0.05.

## 5. Conclusions

Modification of the chemical structure of 2′-hydroxychalcone leads to the formation of a derivatives with various antitumor activity in vitro. Such modification consists of creation the analogues containing a methoxy group instead of a hydroxyl group caused both a reduction and an increase in the antitumor strength of the action depending on the number of groups added and their positions. However, the basic mechanism of action did not change, and all the derivatives obtained exerted antiproliferative and proapoptotic activity and, have the capacity to induce DNA damage, but to varying degrees. The cytotoxic effect of the parent compound and derived derivatives was stronger in relation to cancer cell lines than to normal ones. Further research is needed into the possibility of using chalcones as an adjuvant treatment of canine lymphoma or leukemia.

## Figures and Tables

**Table 1:** IC_50_ values (µM concentration of the tested compounds that inhibits the proliferation of 50% of cells) for all tested compounds and all cell lines used in the study, obtained by the MTT test after 48 h treatment. The results are presented as mean ± standard deviation (SD) of three separate experiments, with three wells each. Statistical differences were analyzed using one-way ANOVA followed by Tukey’s multiple comparison test. Values without common letters (a, b, c, d) in the superscript differ statistically (*p* < 0.05). The table shows differences in the strength of action of each compound on selected cell lines. N.A.—not achieved.

Compound/Cell Line	GL-1	CLBL-1	CNK-89	CLB-70	3T3	J774
2′-hydroxy-2″-methoxychalcone (1)	30.33 ± 5.51 ^ac^	11.88 ± 1.58 ^b^	37.30 ± 6.57 ^a^	24.09 ± 6.23 ^c^	N.A.	37.83 ± 3.40 ^a^
2′-hydroxy-3″-methoxychalcone (2)	33.89 ± 7.00 ^ac^	13.34 ± 3.04 ^b^	42.61 ± 3.60 ^a^	30.66 ± 9.39 ^c^	N.A.	36.50 ± 3.87 ^ac^
2′-hydroxy-4″-methoxychalcone (3)	N.A.	18.78 ± 2.36 ^a^	N.A.	43.81 ± 0.66 ^b^	N.A.	N.A.
2′-hydroxy-3″,4″,5″-trimethoxychalcone (4)	N.A.	15.62 ± 1.52	N.A.	N.A.	N.A.	N.A.
2′-hydroxy-4′,6′,3″,4″,5″-pentamethoxychalcone (5)	N.A.	33.57 ± 9.96	N.A.	N.A.	N.A.	N.A.
2′-hydroxy-2″,5″-dimethoxychalcone (6)	27.52 ± 4.59 ^a^	9.76 ± 0.99 ^b^	40.83 ± 3.00 ^c^	24.47 ± 5.26 ^a^	40.40 ± 3.02 ^c^	29.82 ± 10.04 ^a^
2′-hydroxy-4′,6′-dimethoxychalcone (7)	31.18 ± 6.32 ^ac^	9.18 ± 0.75 ^b^	38.63 ± 8.05 ^ad^	26.78 ± 9.03 ^c^	46.11 ± 4.66 ^d^	32.17 ± 8.69 ^ac^
2′-hydroxychalcone (8)	40.58 ± 3.92 ^a^	8.04 ± 1.08 ^b^	48.51 ± 1.52 ^c^	38.94 ± 6.02 ^a^	N.A.	N.A.

## Supplementary Materials

The following are available online. Figure S1: ^1^H NMR spectral of 2′-hydroxy-2″-methoxychalcone (**1**) (CDCl3, 600 MHz), Figure S2: Part of the ^1^H NMR spectral 2′-hydroxy-2″-methoxychalcone (**1**) (CDCl3, 600 MHz), Figure S3: ^13^C NMR spectral of 2′-hydroxy-2″-methoxychalcone (**1**) (CDCl3, 151 MHz), Figure S4: COSY spectral of 2′-hydroxy-2″-methoxychalcone (**1**) (CDCl3, 151 MHz), Figure S5: HSQC spectral of 2′-hydroxy-2″-methoxychalcone (**1**) (CDCl3, 151 MHz), Figure S6: ^1^H NMR spectral of 2′-hydroxy-3″-methoxychalcone (**2**) (CDCl3, 600 MHz), Figure S7: Part of the ^1^H NMR spectral 2′-hydroxy-3″-methoxychalcone (**2**) (CDCl3, 600 MHz), Figure S8: ^13^C NMR spectral of 2′-hydroxy-3″-methoxychalcone (**2**) (CDCl3, 151 MHz), Figure S9: HSQC spectral of 2′-hydroxy-3″-methoxychalcone (**2**) (CDCl3, 151 MHz), Figure S10: ^1^H NMR spectral of 2′-hydroxy-4″-methoxychalcone (**3**) (CDCl3, 600 MHz), Figure S11: Part of the ^1^H NMR spectral 2′-hydroxy-4″-methoxychalcone (**3**) (CDCl3, 600 MHz), Figure S12: ^13^C NMR spectral of 2′-hydroxy-4″-methoxychalcone (**3**) (CDCl3, 151 MHz), Figure S13: COSY spectral of 2′-hydroxy-4″-methoxychalcone (**3**) (CDCl3, 151 MHz), Figure S14: HSQC spectral of 2′-hydroxy-4″-methoxychalcone (**3**) (CDCl3, 151 MHz), Figure S15: ^1^H NMR spectral of 2′-hydroxy-3″,4″,5″-trimethoxychalcone (**4**) (CDCl3, 600 MHz), Figure S16: Part of the ^1^H NMR spectral 2′-hydroxy-3″,4″,5″-trimethoxychalcone (**4**) (CDCl3, 600 MHz), Figure S17: ^13^C NMR spectral of 2′-hydroxy-3″,4″,5″-trimethoxychalcone (**4**) (CDCl3, 151 MHz), Figure S18: COSY spectral of 2′-hydroxy-3″,4″,5″-trimethoxychalcone (**4**) (CDCl3, 151 MHz), Figure S19: HSQC spectral of 2′-hydroxy-3″,4″,5″-trimethoxychalcone (**4**) (CDCl3, 151 MHz), Figure S20: ^1^H NMR spectral of 2′-hydroxy-4′,6′,3″,4″,5″-pentamethoxychalcone (**5**) (CDCl3, 600 MHz), Figure S21: Part of the ^1^H NMR spectral 2′-hydroxy-4′,6′,3″,4″,5″-pentamethoxychalcone (**5**) (CDCl3, 600 MHz), Figure S22: ^13^C NMR spectral of 2′-hydroxy-4′,6′,3″,4″,5″-pentamethoxychalcone (**5**) (CDCl3, 151 MHz), Figure S23: HSQC spectral of 2′-hydroxy-4′,6′,3″,4″,5″-pentamethoxychalcone (**5**) (CDCl3, 151 MHz), Figure S24 HMBC spectral of 2′-hydroxy-4′,6′,3″,4″,5″-pentamethoxychalcone (**5**) (CDCl3, 151 MHz), Figure S25: ^1^H NMR spectral of 2′-hydroxy-2″,5″-dimethoxychalcone (**6**) (CDCl3, 600 MHz), Figure S26: Part of the ^1^H NMR spectral 2′-hydroxy-2″,5″-dimethoxychalcone (**6**) (CDCl3, 600 MHz), Figure S27: ^13^C NMR spectral of 2′-hydroxy-2″,5″-dimethoxychalcone (**6**) (CDCl3, 151 MHz), Figure S28: COSY spectral of 2′-hydroxy-2″,5″-dimethoxychalcone (**6**) (CDCl3, 151 MHz), Figure S29: HSQC spectral of 2′-hydroxy-2″,5″-dimethoxychalcone (**6**) (CDCl3, 151 MHz).
