# Supplementary material for: Chalcone Methoxy Derivatives Exhibit Antiproliferative and Proapoptotic Activity on Canine Lymphoma and Leukemia Cells
Source: Molecules. 2020 Sep 23;25(19):4362. doi: 10.3390/molecules25194362 (PMC7582533; doi:10.3390/molecules25194362)
Supplement: Supplementary file 1 [file molecules-25-04362-s001.pdf]

Supplementary Data

Chalcone Methoxy Derivatives Exhibit Antiproliferative and Proapoptotic Activity on Canine Lymphoma and Leukemia Cells

Aleksandra Pawlak [1,*], Marta Henklewska [1], Beatriz Hernández Suárez [1], Mateusz Łużny [2], Ewa Kozłowska [2], Bożena Obmińska-Mrukowicz [1] and Tomasz Janeczko [2]

[1] Department of Pharmacology and Toxicology, Wrocław University of Environmental and Life Sciences, C.K. Norwida 31, 50-375 Wrocław, Poland; marta.henklewska@upwr.edu.pl (M.H.); beatriz.hernandez-suarez@upwr.edu.pl (B.H.S.); b.mrukowicz@gmail.com (B.O.-M.)

[2] Department of Chemistry, Wrocław University of Environmental and Life Sciences, Norwida 25, 50-375 Wrocław, Poland; mateusz.luzny@upwr.edu.pl (M.Ł.); ewa.kozlowska1@upwr.edu.pl (E.K.); tomasz.janeczko@upwr.edu.pl (T.J.)

* Correspondence: aleksandra.pawlak@upwr.edu.pl

Content

Figure S1. ^{1}H NMR spectral of 2′-hydroxy-2″-methoxychalcone (**1**) ($CDCl_3$, 600 MHz).
Figure S2. Part of the ^{1}H NMR spectral 2′-hydroxy-2″-methoxychalcone (**1**) ($CDCl_3$, 600 MHz).
Figure S3. ^{13}C NMR spectral of 2′-hydroxy-2″-methoxychalcone (**1**) ($CDCl_3$, 151 MHz).
Figure S4. COSY spectral of 2′-hydroxy-2″-methoxychalcone (**1**) ($CDCl_3$, 151 MHz).
Figure S5. HSQC spectral of 2′-hydroxy-2″-methoxychalcone (**1**) ($CDCl_3$, 151 MHz).
Figure S6. ^{1}H NMR spectral of 2′-hydroxy-3″-methoxychalcone (**2**) ($CDCl_3$, 600 MHz).
Figure S7. Part of the ^{1}H NMR spectral 2′-hydroxy-3″-methoxychalcone (**2**) ($CDCl_3$, 600 MHz).
Figure S8. ^{13}C NMR spectral of 2′-hydroxy-3″-methoxychalcone (**2**) ($CDCl_3$, 151 MHz).
Figure S9. HSQC spectral of 2′-hydroxy-3″-methoxychalcone (**2**) ($CDCl_3$, 151 MHz).
Figure S10. ^{1}H NMR spectral of 2′-hydroxy-4″-methoxychalcone (**3**) ($CDCl_3$, 600 MHz).
Figure S11. Part of the ^{1}H NMR spectral 2′-hydroxy-4″-methoxychalcone (**3**) ($CDCl_3$, 600 MHz).
Figure S12. ^{13}C NMR spectral of 2′-hydroxy-4″-methoxychalcone (**3**) ($CDCl_3$, 151 MHz).
Figure S13. COSY spectral of 2′-hydroxy-4″-methoxychalcone (**3**) ($CDCl_3$, 151 MHz).
Figure S14. HSQC spectral of 2′-hydroxy-4″-methoxychalcone (**3**) ($CDCl_3$, 151 MHz).
Figure S15. ^{1}H NMR spectral of 2′-hydroxy-3″,4″,5″-trimethoxychalcone (**4**) ($CDCl_3$, 600 MHz).
Figure S16. Part of the ^{1}H NMR spectral 2′-hydroxy-3″,4″,5″-trimethoxychalcone (**4**) ($CDCl_3$, 600 MHz).
Figure S17. ^{13}C NMR spectral of 2′-hydroxy-3″,4″,5″-trimethoxychalcone (**4**) ($CDCl_3$, 151 MHz).
Figure S18. COSY spectral of 2′-hydroxy-3″,4″,5″-trimethoxychalcone (**4**) ($CDCl_3$, 151 MHz).
Figure S19. HSQC spectral of 2′-hydroxy-3″,4″,5″-trimethoxychalcone (**4**) ($CDCl_3$, 151 MHz).
Figure S20. ^{1}H NMR spectral of 2′-hydroxy-4′,6′,3″,4″,5″-pentamethoxychalcone (**5**) ($CDCl_3$, 600 MHz).
Figure S21. Part of the ^{1}H NMR spectral 2′-hydroxy-4′,6′,3″,4″,5″-pentamethoxychalcone (**5**) ($CDCl_3$, 600 MHz).
Figure S22. ^{13}C NMR spectral of 2′-hydroxy-4′,6′,3″,4″,5″-pentamethoxychalcone (**5**) ($CDCl_3$, 151 MHz).
Figure S23. HSQC spectral of 2′-hydroxy-4′,6′,3″,4″,5″-pentamethoxychalcone (**5**) ($CDCl_3$, 151 MHz).
Figure S24. HMBC spectral of 2′-hydroxy-4′,6′,3″,4″,5″-pentamethoxychalcone (**5**) ($CDCl_3$, 151 MHz).
Figure S25. ^{1}H NMR spectral of 2′-hydroxy-2″,5″-dimethoxychalcone (**6**) ($CDCl_3$, 600 MHz).
Figure S26. Part of the ^{1}H NMR spectral 2′-hydroxy-2″,5″-dimethoxychalcone (**6**) ($CDCl_3$, 600 MHz).
Figure S27. ^{13}C NMR spectral of 2′-hydroxy-2″,5″-dimethoxychalcone (**6**) ($CDCl_3$, 151 MHz).
Figure S28. COSY spectral of 2′-hydroxy-2″,5″-dimethoxychalcone (**6**) ($CDCl_3$, 151 MHz).
Figure S29. HSQC spectral of 2′-hydroxy-2″,5″-dimethoxychalcone (**6**) ($CDCl_3$, 151 MHz).

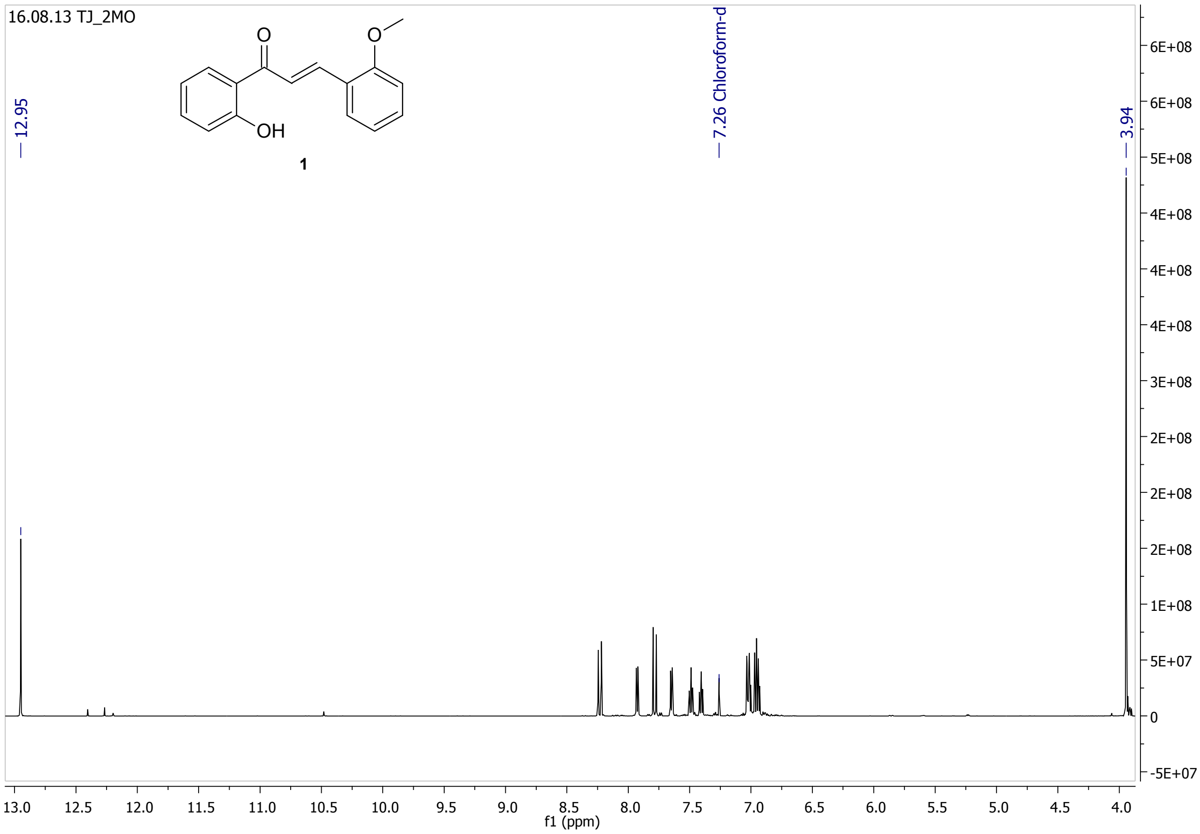

Figure S1. ^{1}H NMR spectral of 2′-hydroxy-2″-methoxychalcone (**1**) ($CDCl_3$, 600 MHz).

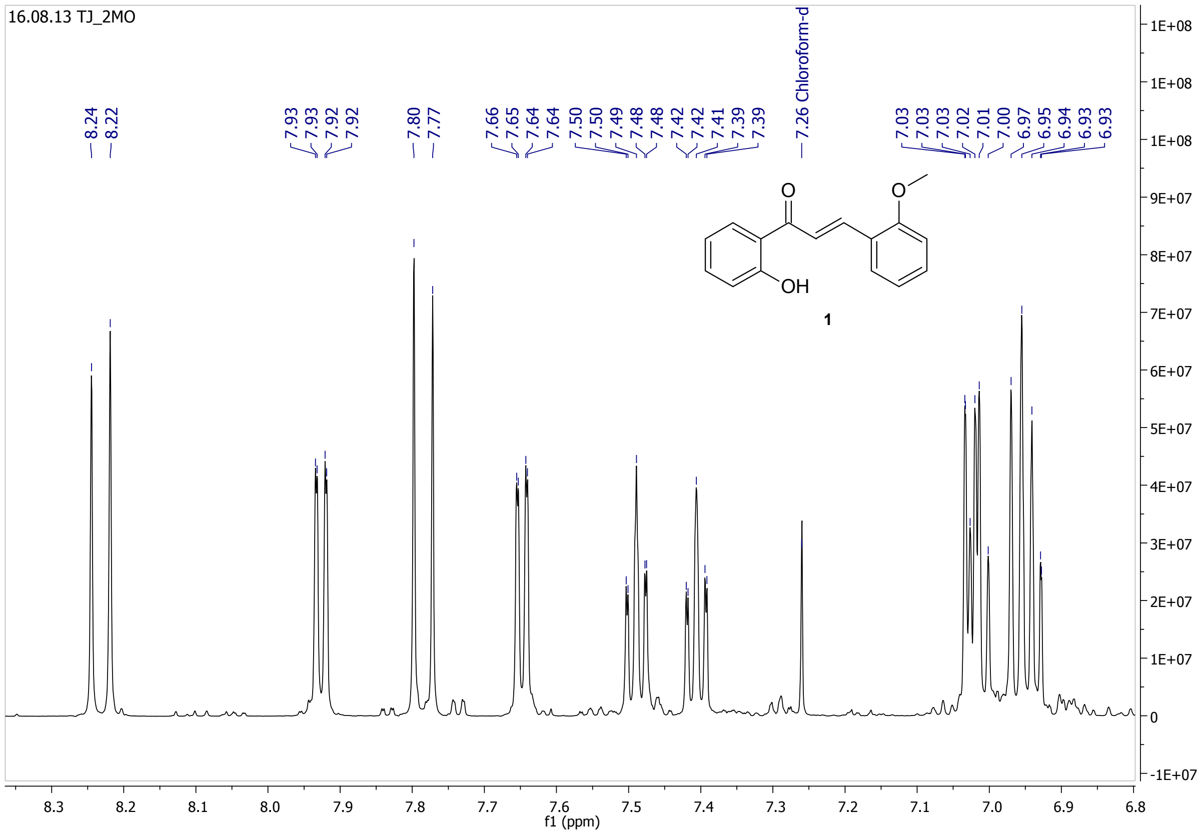

Figure S2. Part of the ^{1}H NMR spectral 2′-hydroxy-2″-methoxychalcone (**1**) ($CDCl_3$, 600 MHz).

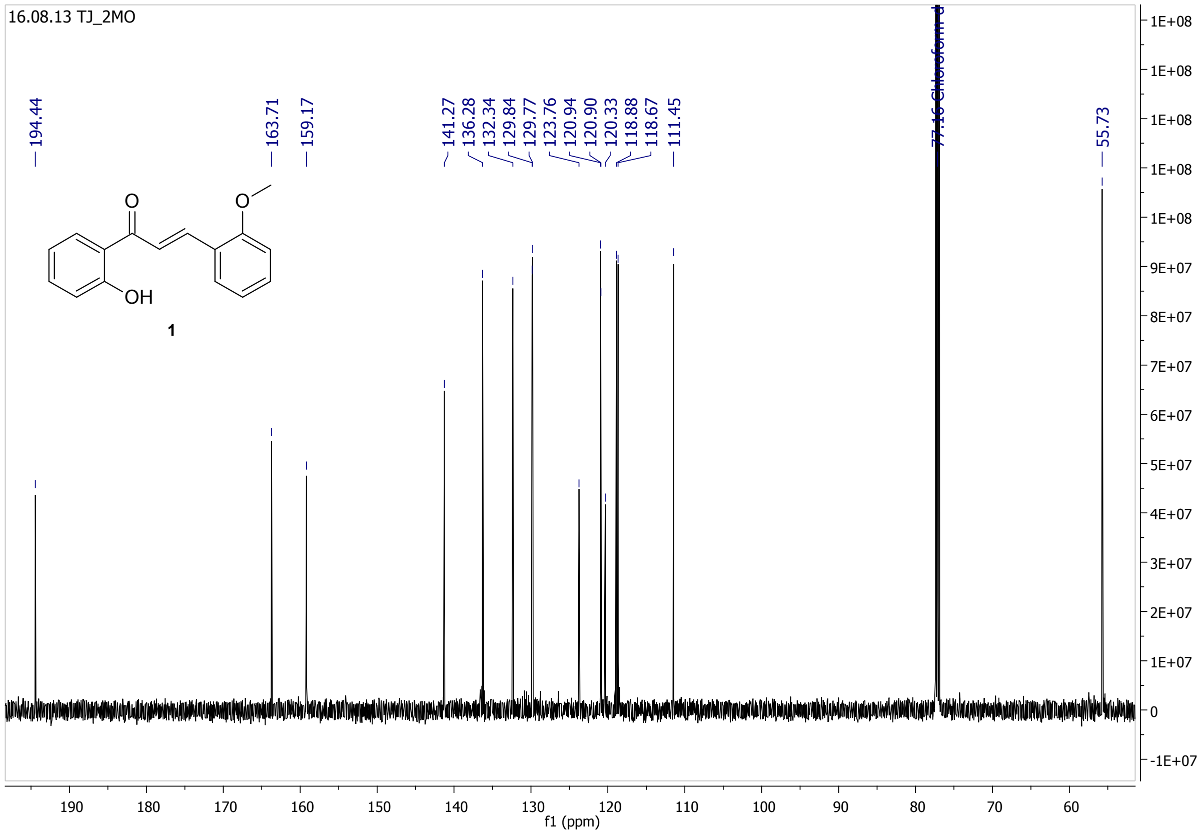

Figure S3. ^{13}C NMR spectral of 2′-hydroxy-2″-methoxychalcone (**1**) ($CDCl_3$, 151 MHz).

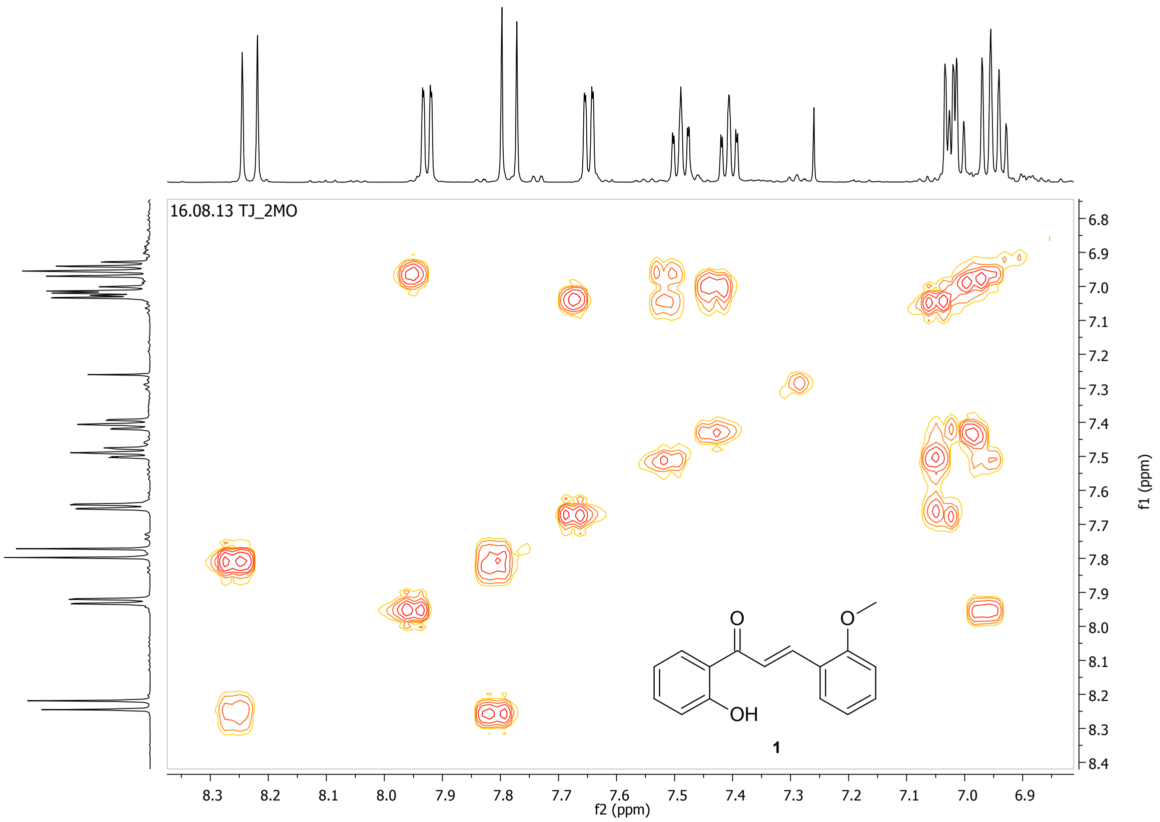

Figure S4. COSY spectral of 2′-hydroxy-2″-methoxychalcone (**1**) ($CDCl_3$, 151 MHz).

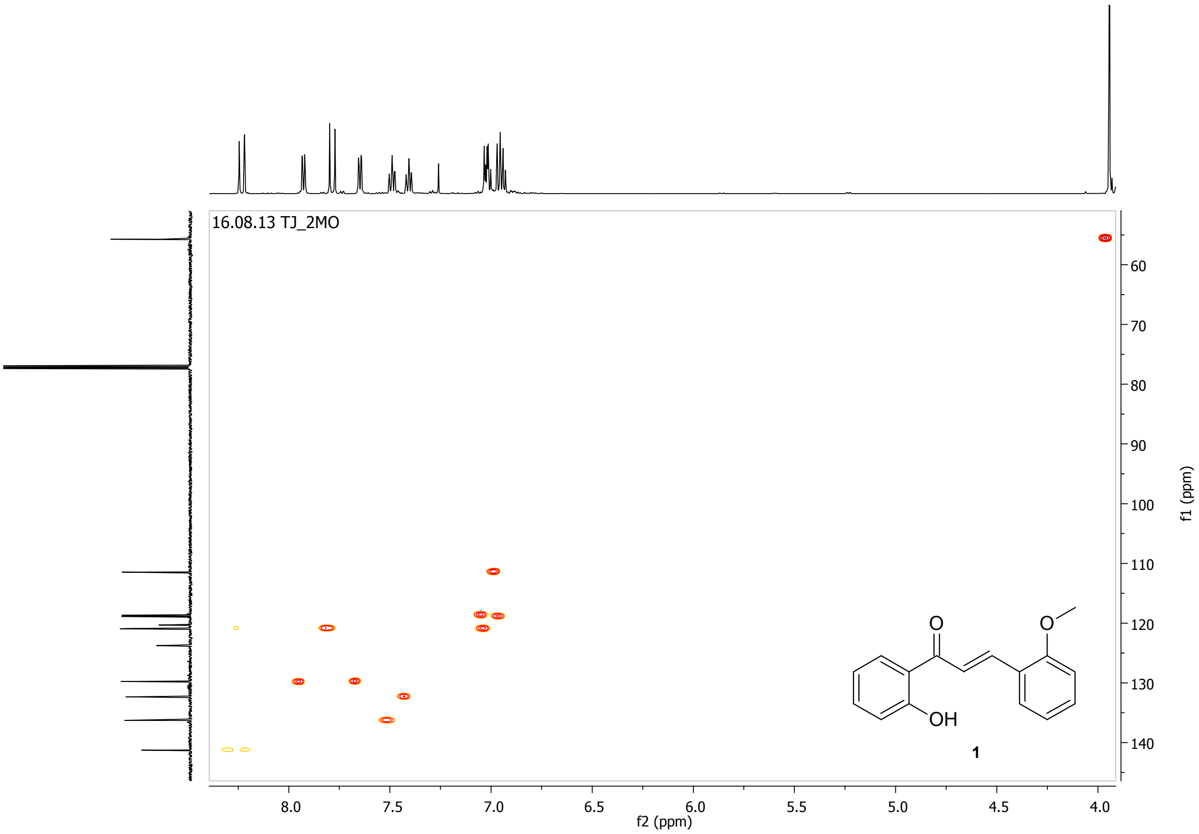

Figure S5. HSQC spectral of 2′-hydroxy-2″-methoxychalcone (**1**) ($CDCl_3$, 151 MHz).

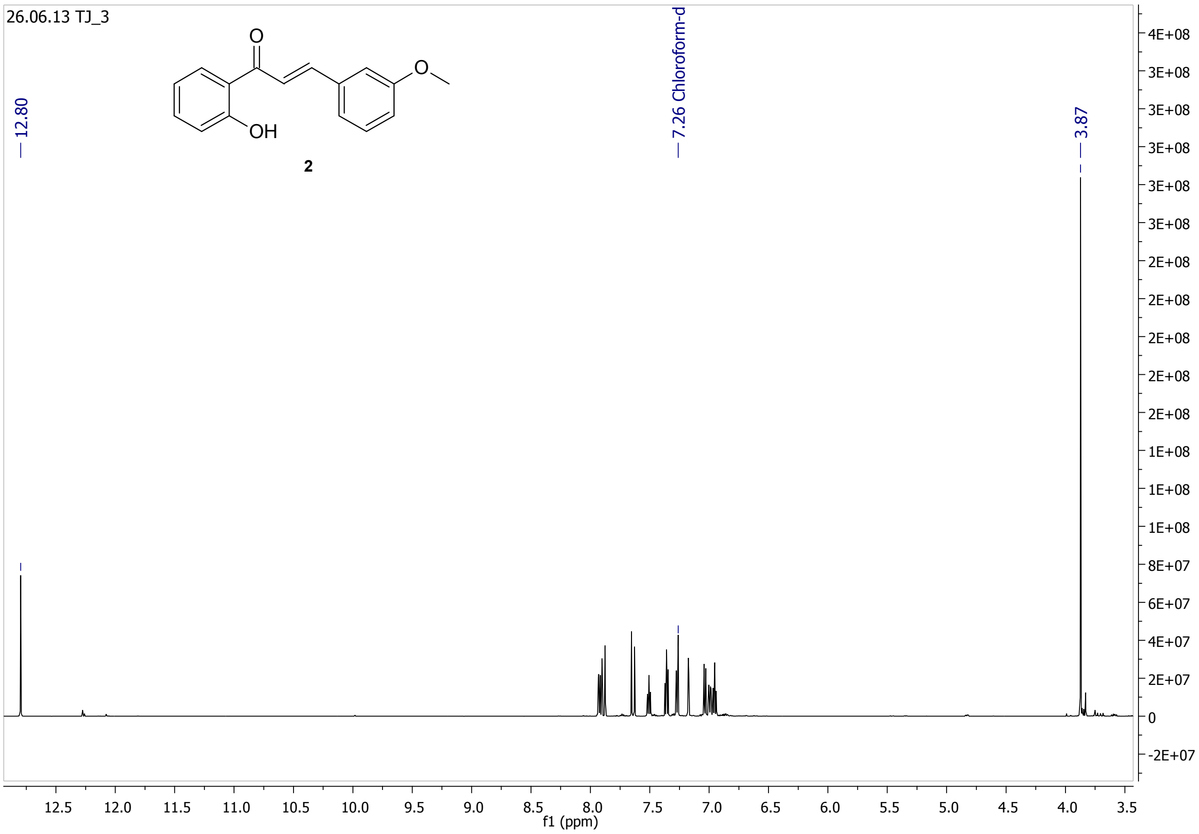

Figure S6. ^{1}H NMR spectral of 2′-hydroxy-3″-methoxychalcone (**2**) ($CDCl_3$, 600 MHz).

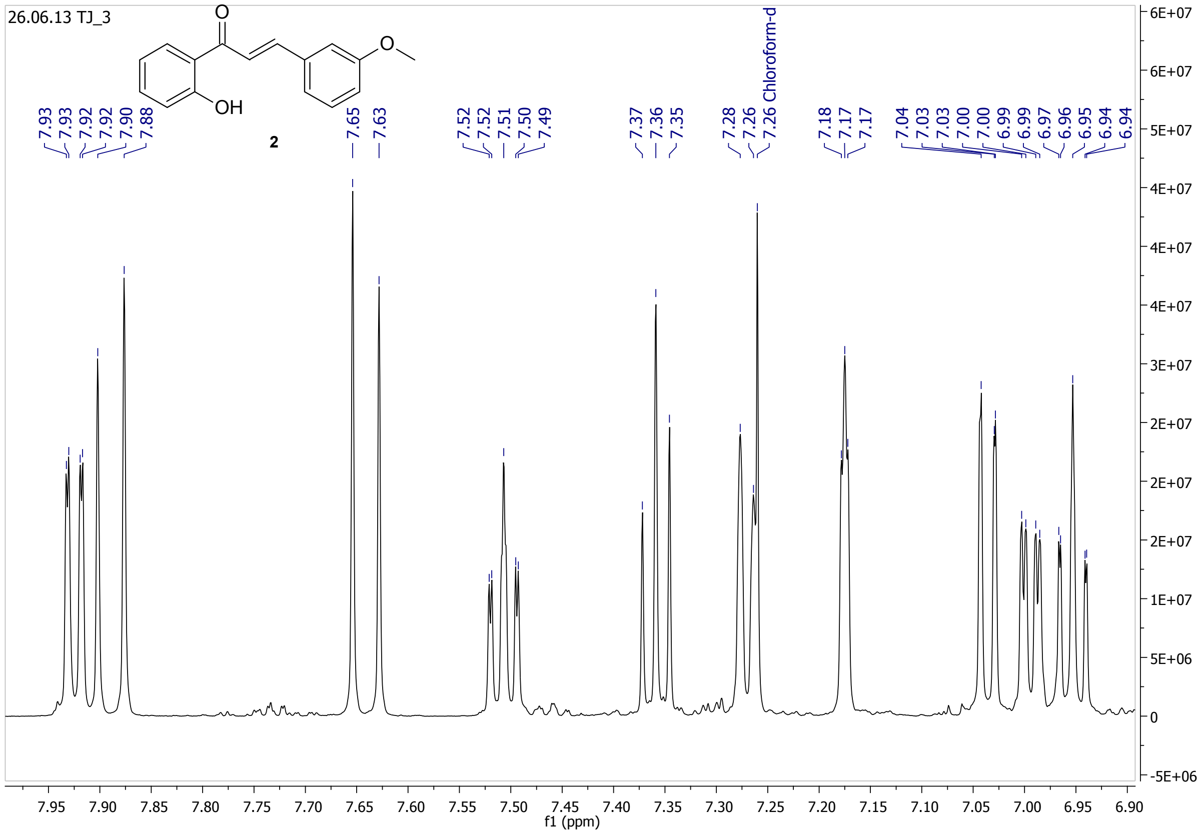

Figure S7. Part of the ^{1}H NMR spectral 2′-hydroxy-3″-methoxychalcone (**2**) ($CDCl_3$, 600 MHz).

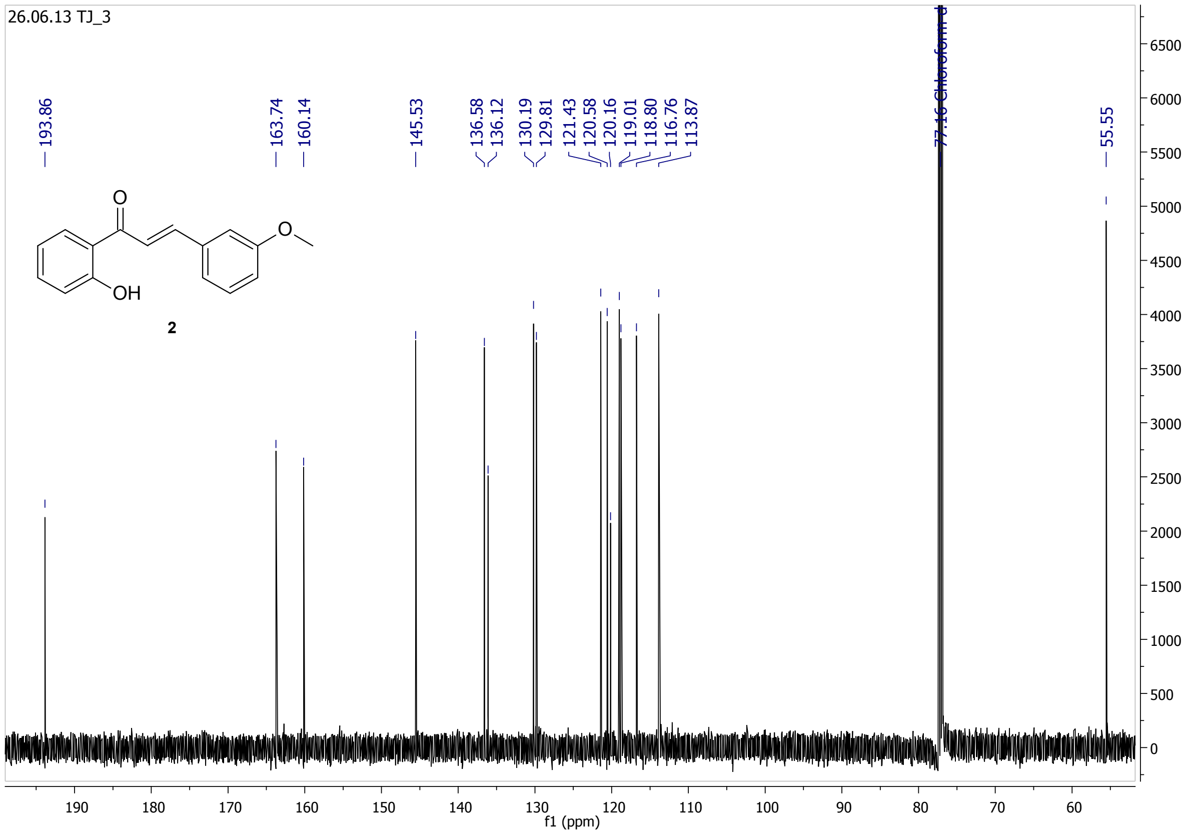

Figure S8. ^{13}C NMR spectral of 2′-hydroxy-3″-methoxychalcone (**2**) ($CDCl_3$, 151 MHz).

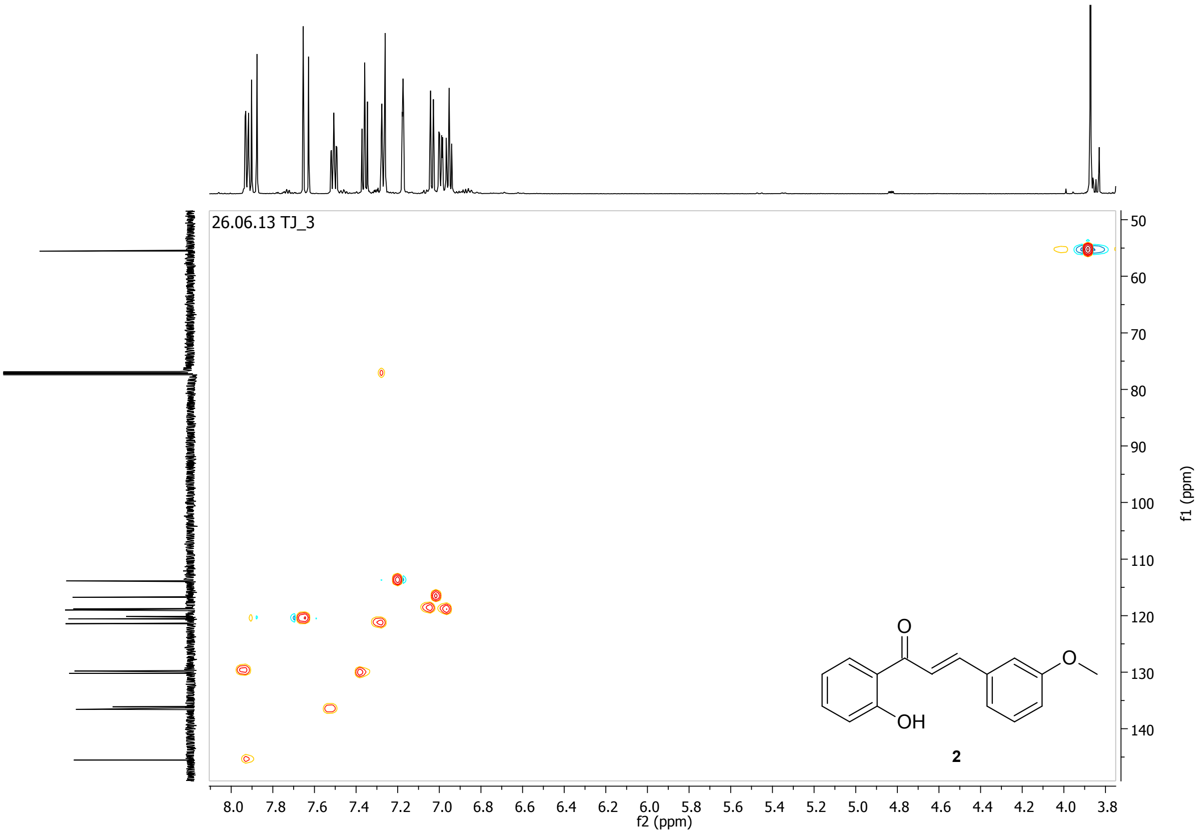

Figure S9. HSQC spectral of 2′-hydroxy-3″-methoxychalcone (**2**) ($CDCl_3$, 151 MHz).

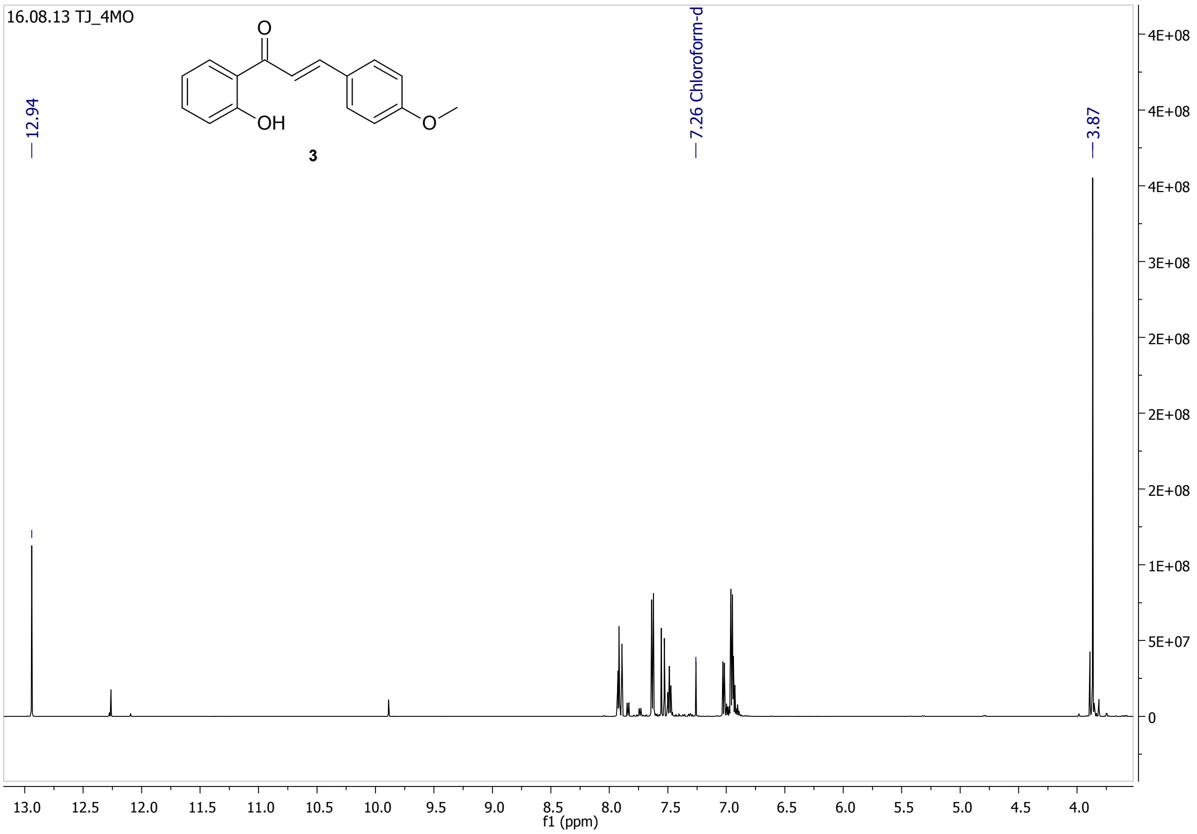

Figure S10. ^{1}H NMR spectral of 2′-hydroxy-4″-methoxychalcone (**3**) ($CDCl_3$, 600 MHz).

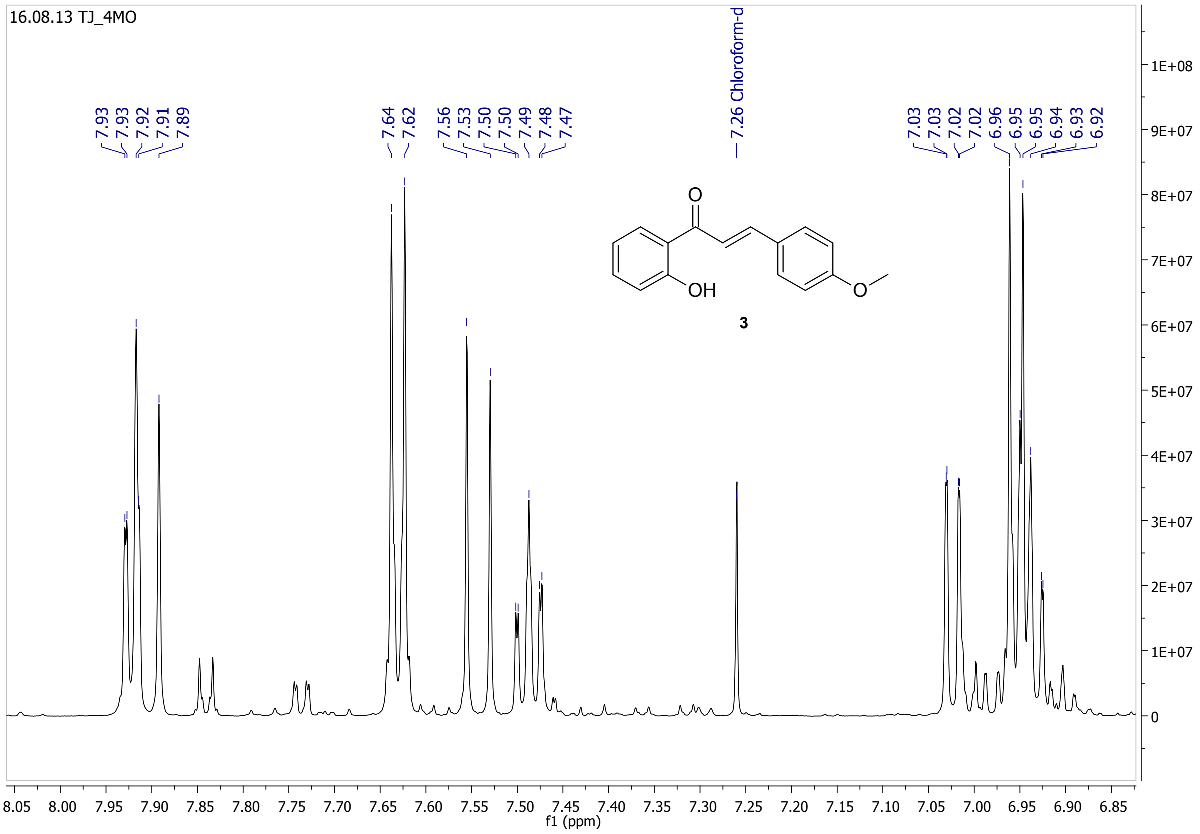

Figure S11. Part of the ^{1}H NMR spectral 2′-hydroxy-4″-methoxychalcone (**3**) ($CDCl_3$, 600 MHz).

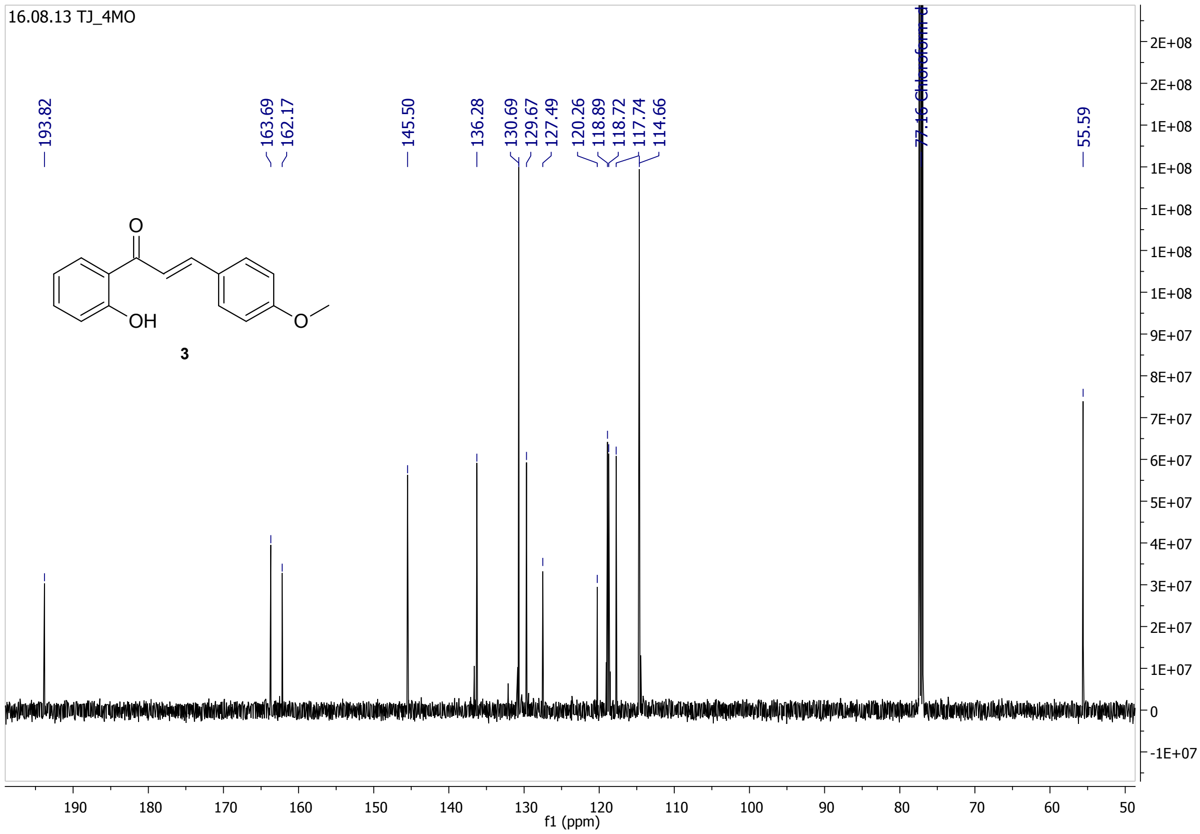

Figure S12. ^{13}C NMR spectral of 2′-hydroxy-4″-methoxychalcone (**3**) ($CDCl_3$, 151 MHz).

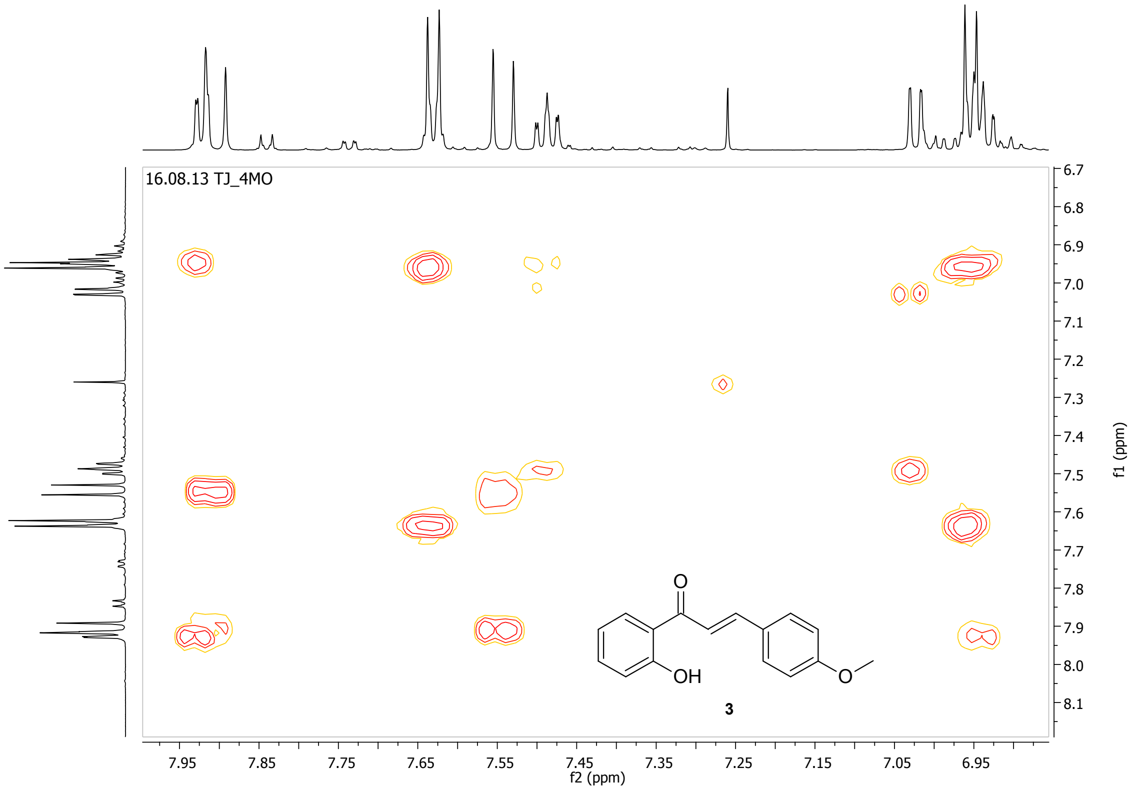

Figure S13. COSY spectral of 2′-hydroxy-4″-methoxychalcone (**3**) ($CDCl_3$, 151 MHz).

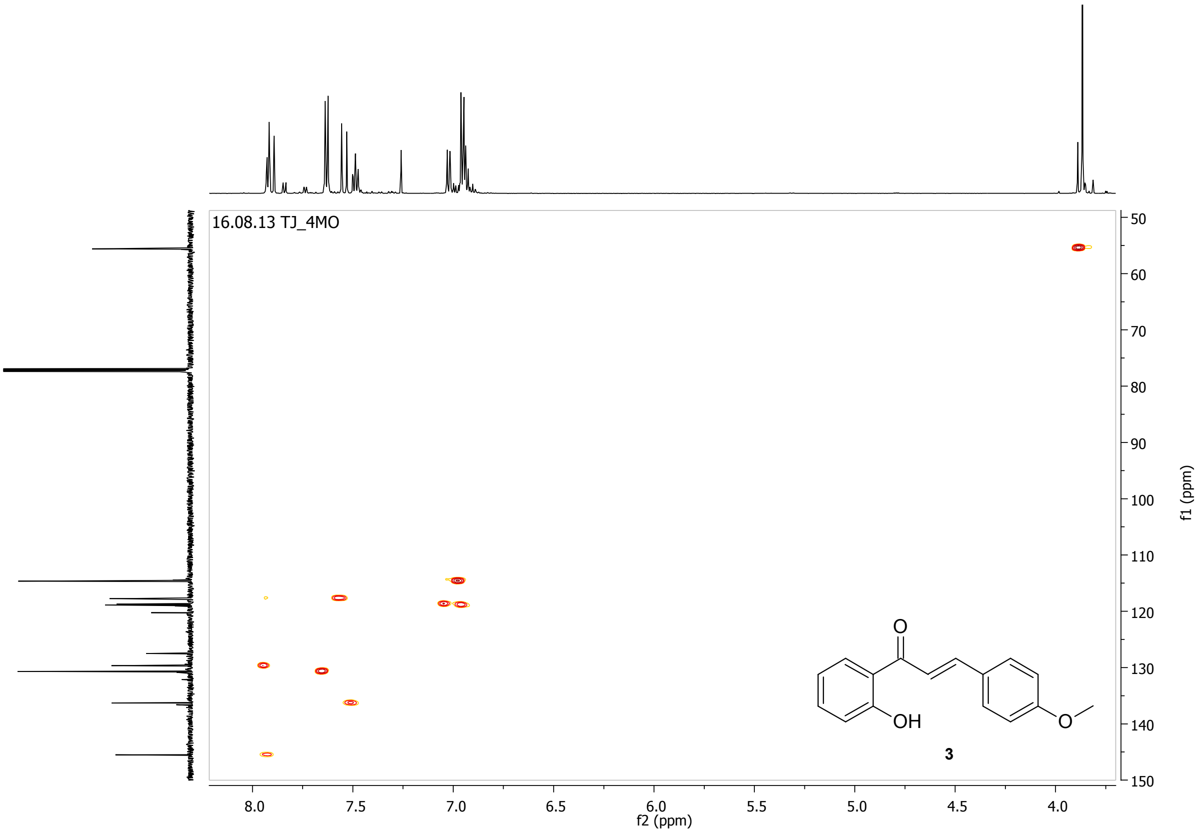

Figure S14. HSQC spectral of 2′-hydroxy-4″-methoxychalcone (**3**) ($CDCl_3$, 151 MHz).

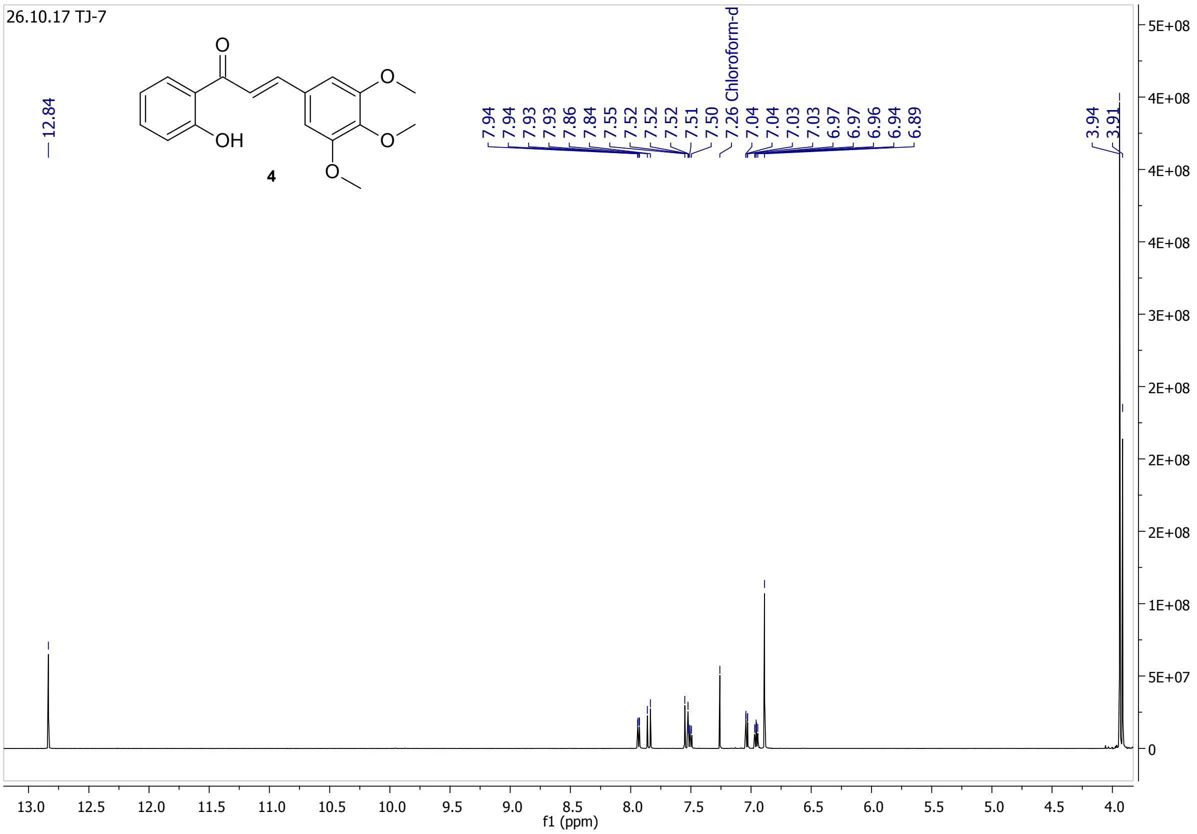

Figure S15. ^{1}H NMR spectral of 2′-hydroxy-3″,4″,5″-trimethoxychalcone (**4**) ($CDCl_3$, 600 MHz).

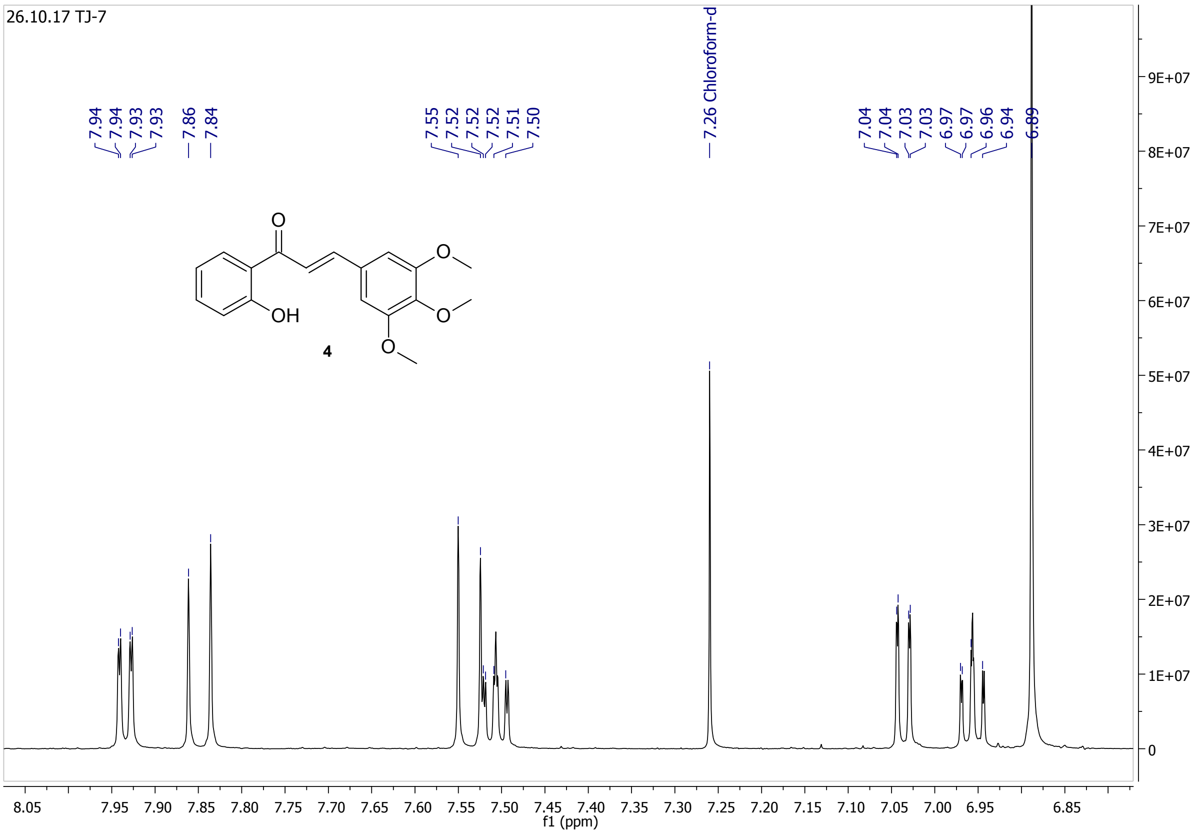

Figure S16. Part of the ^{1}H NMR spectral 2′-hydroxy-3″,4″,5″-trimethoxychalcone (**4**) ($CDCl_3$, 600 MHz).

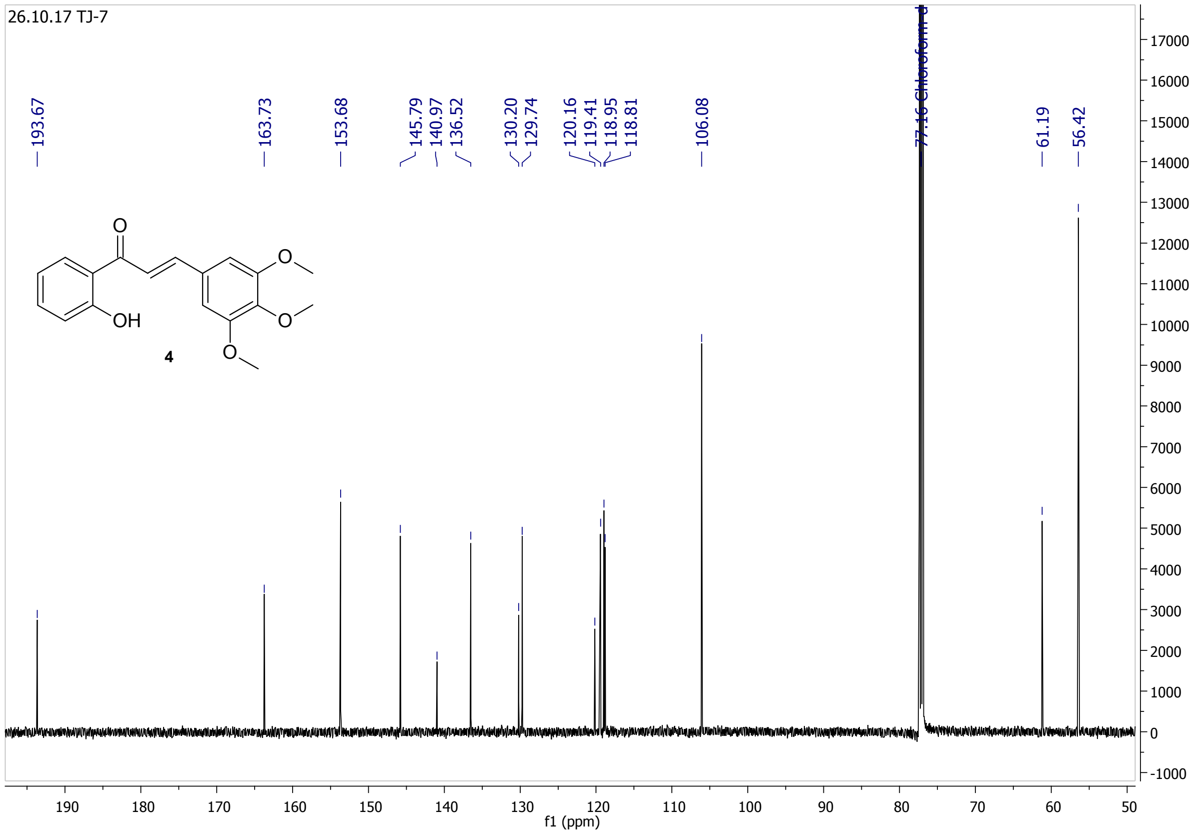

Figure S17. ^{13}C NMR spectral of 2′-hydroxy-3″,4″,5″-trimethoxychalcone (**4**) ($CDCl_3$, 151 MHz).

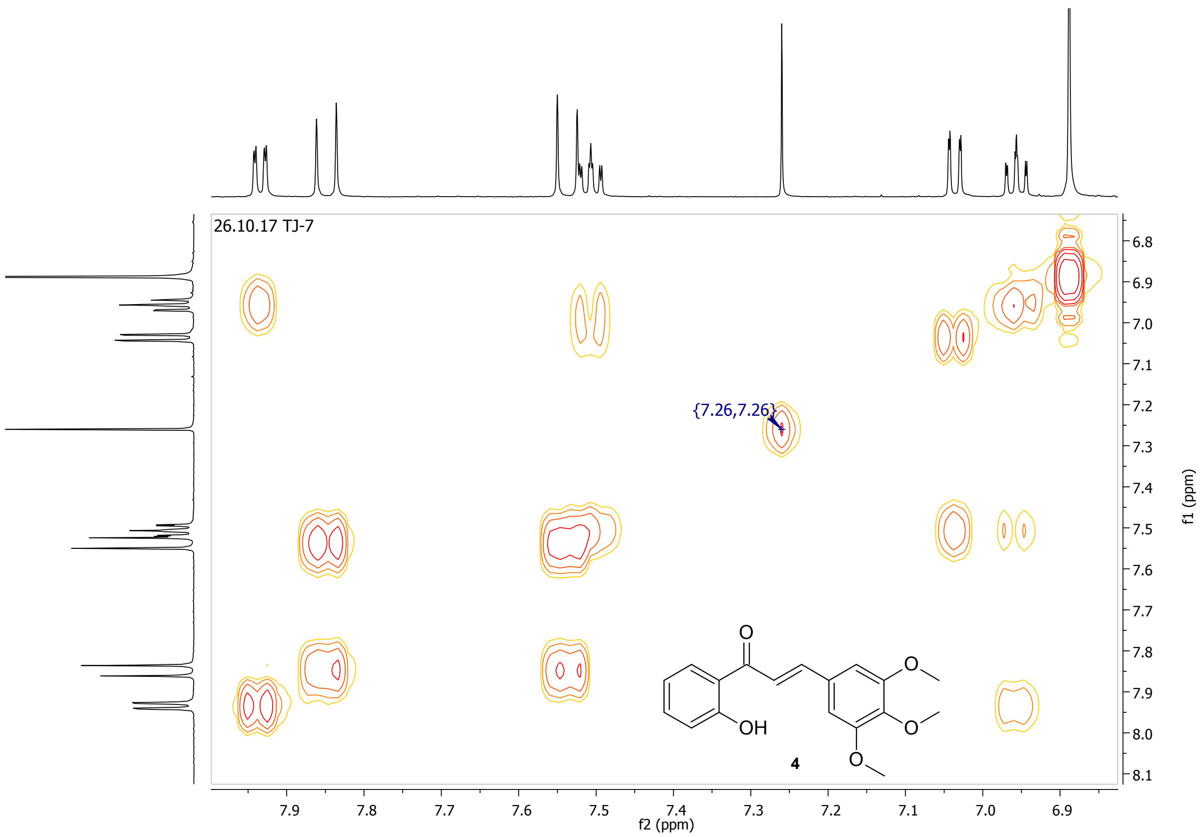

Figure S18. COSY spectral of 2′-hydroxy-3″,4″,5″-trimethoxychalcone (**4**) ($CDCl_3$, 151 MHz).

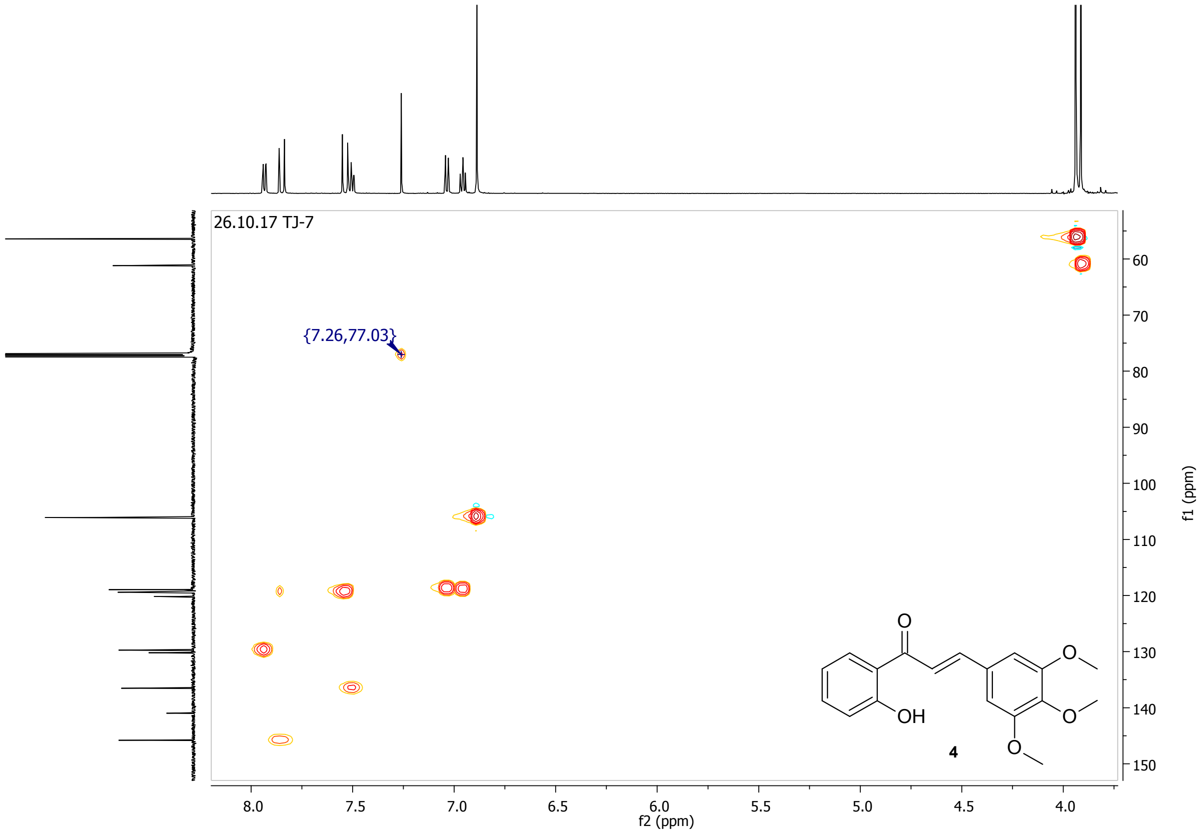

Figure S19. HSQC spectral of 2′-hydroxy-3″,4″,5″-trimethoxychalcone (**4**) ($CDCl_3$, 151 MHz).

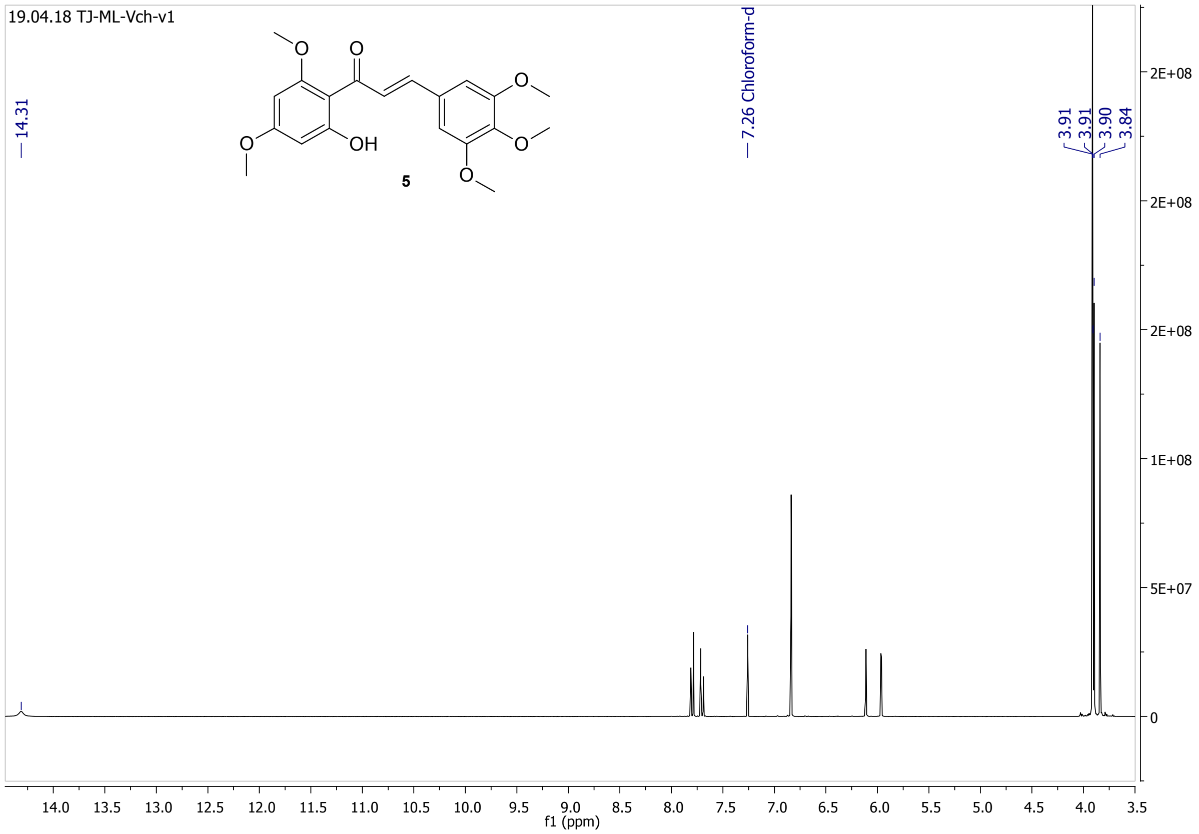

Figure S20. ^{1}H NMR spectral of 2′-hydroxy-4′,6′,3″,4″,5″-pentamethoxychalcone (**5**) ($CDCl_3$, 600 MHz).

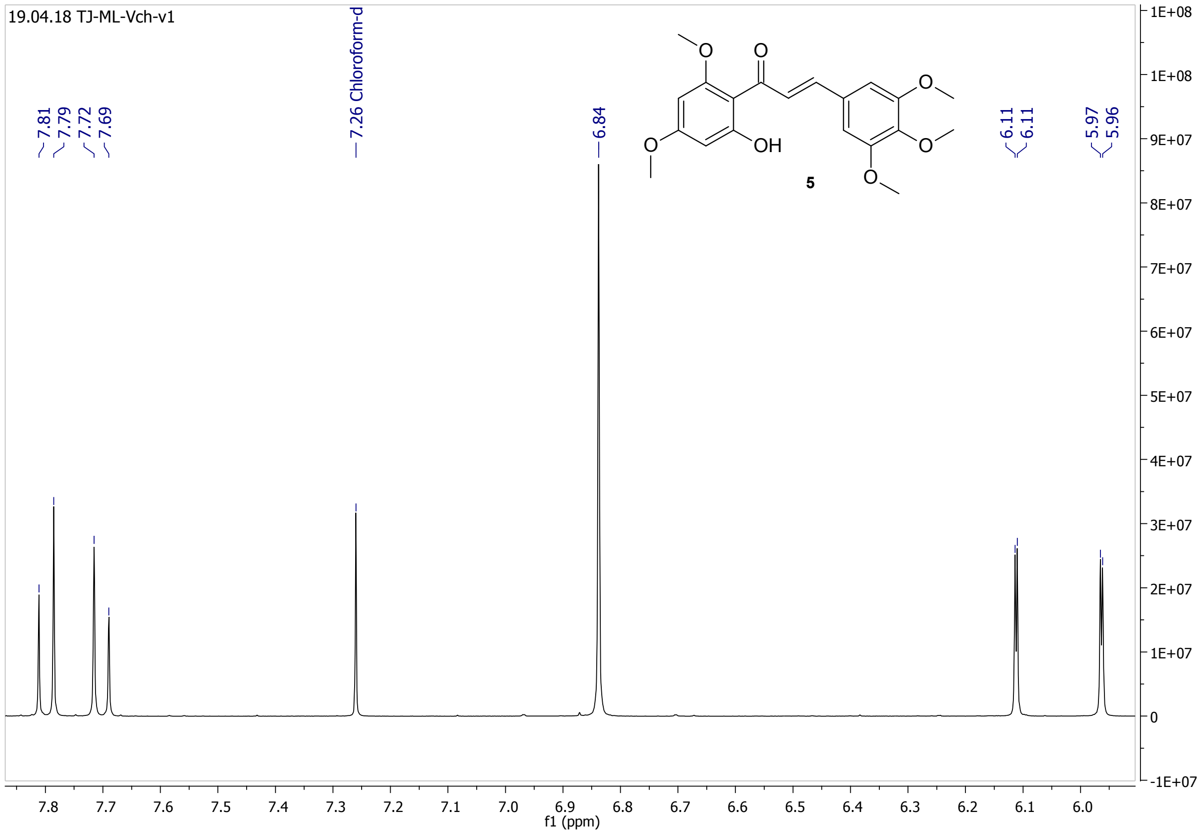

Figure S21. Part of the ^{1}H NMR spectral 2′-hydroxy-4′,6′,3″,4″,5″-pentamethoxychalcone (**5**) ($CDCl_3$, 600 MHz).

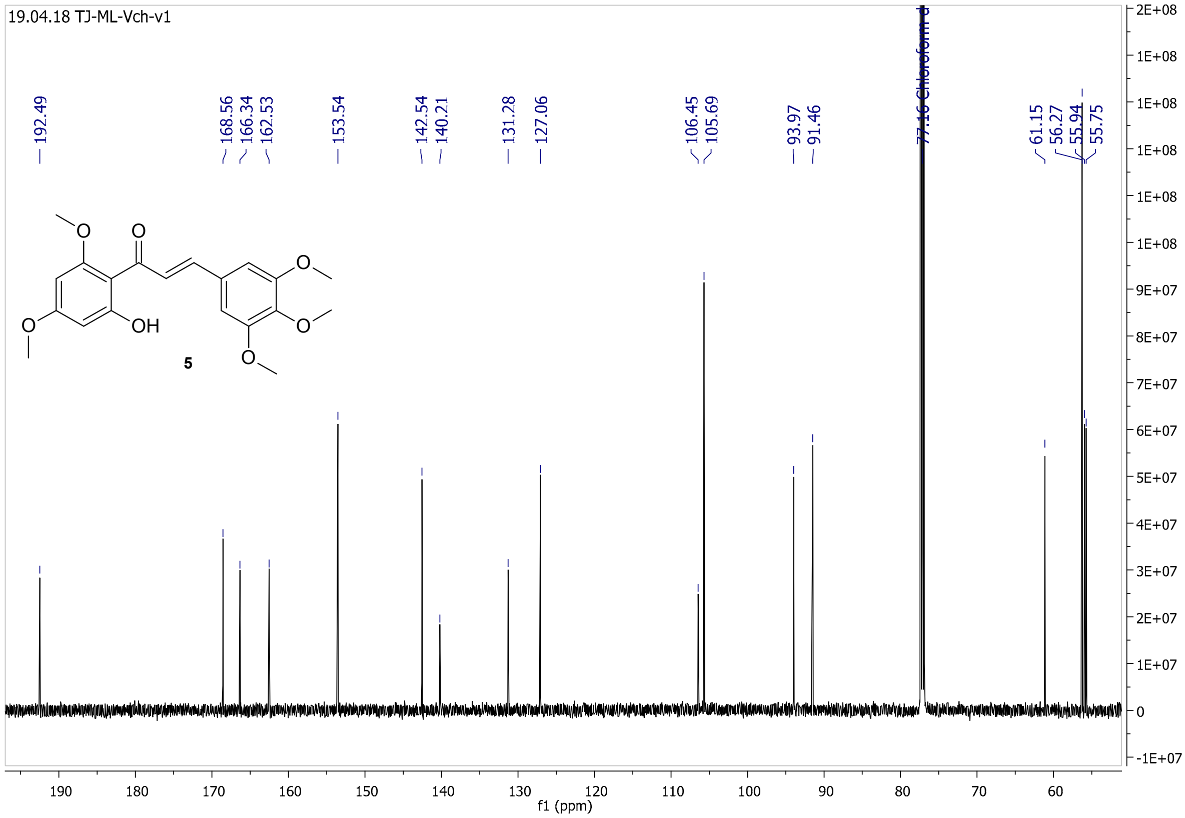

Figure S22. ^{13}C NMR spectral of 2′-hydroxy-4′,6′,3″,4″,5″-pentamethoxychalcone (**5**) ($CDCl_3$, 151 MHz).

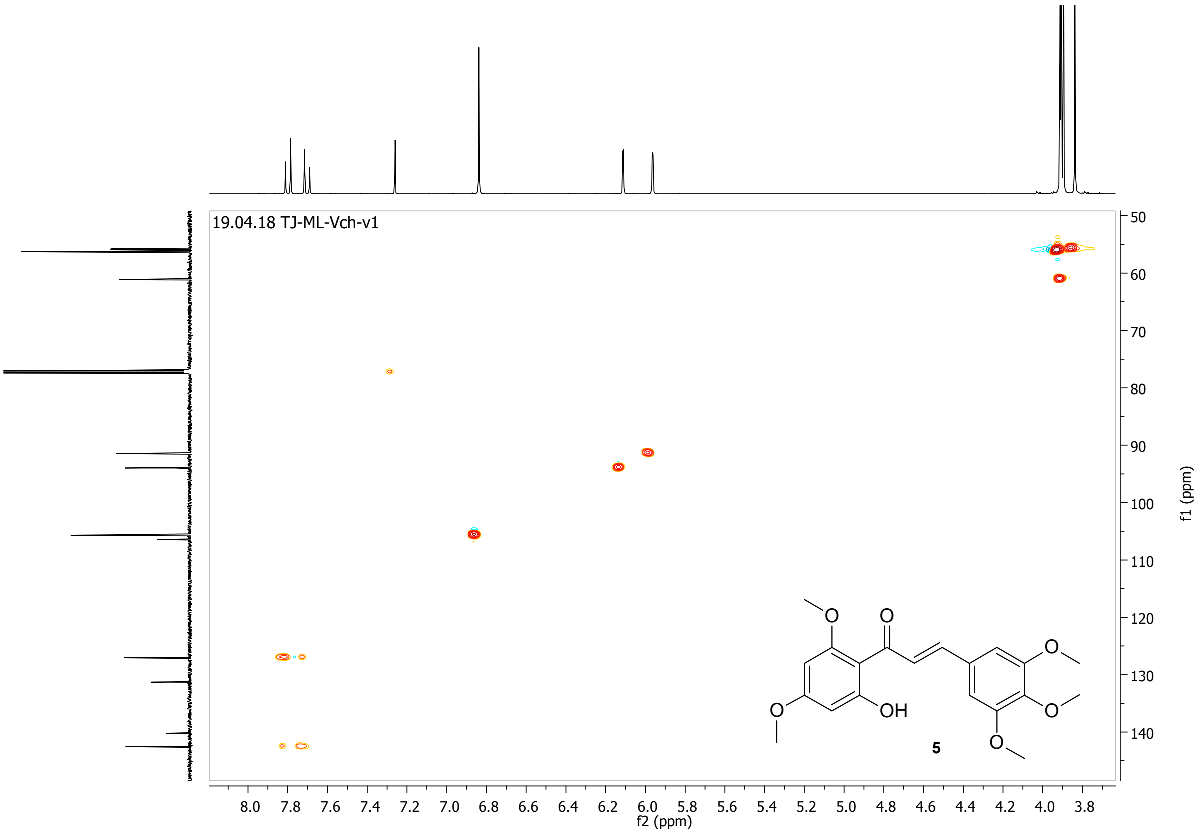

Figure S23. HSQC spectral of 2′-hydroxy-4′,6′,3″,4″,5″-pentamethoxychalcone (**5**) ($CDCl_3$, 151 MHz).

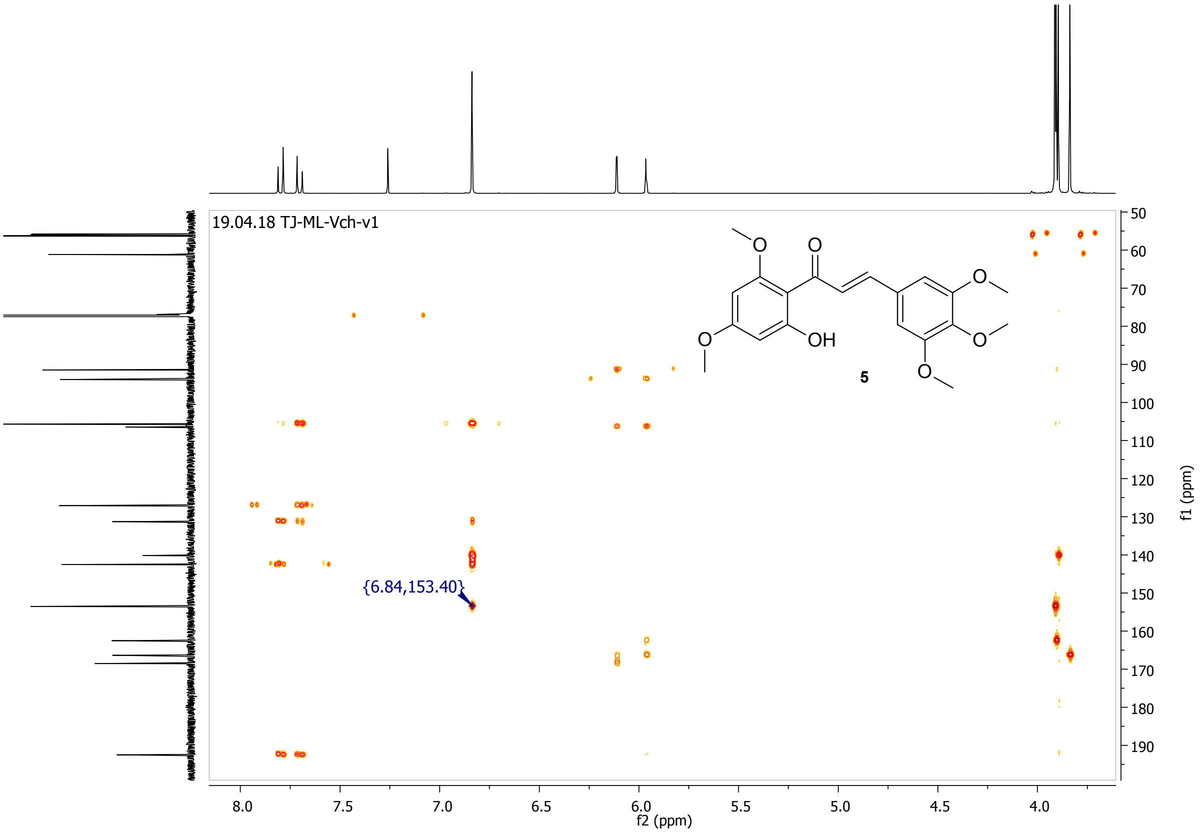

Figure S24. HMBC spectral of 2′-hydroxy-4′,6′,3″,4″,5″-pentamethoxychalcone (**5**) ($CDCl_3$, 151 MHz).

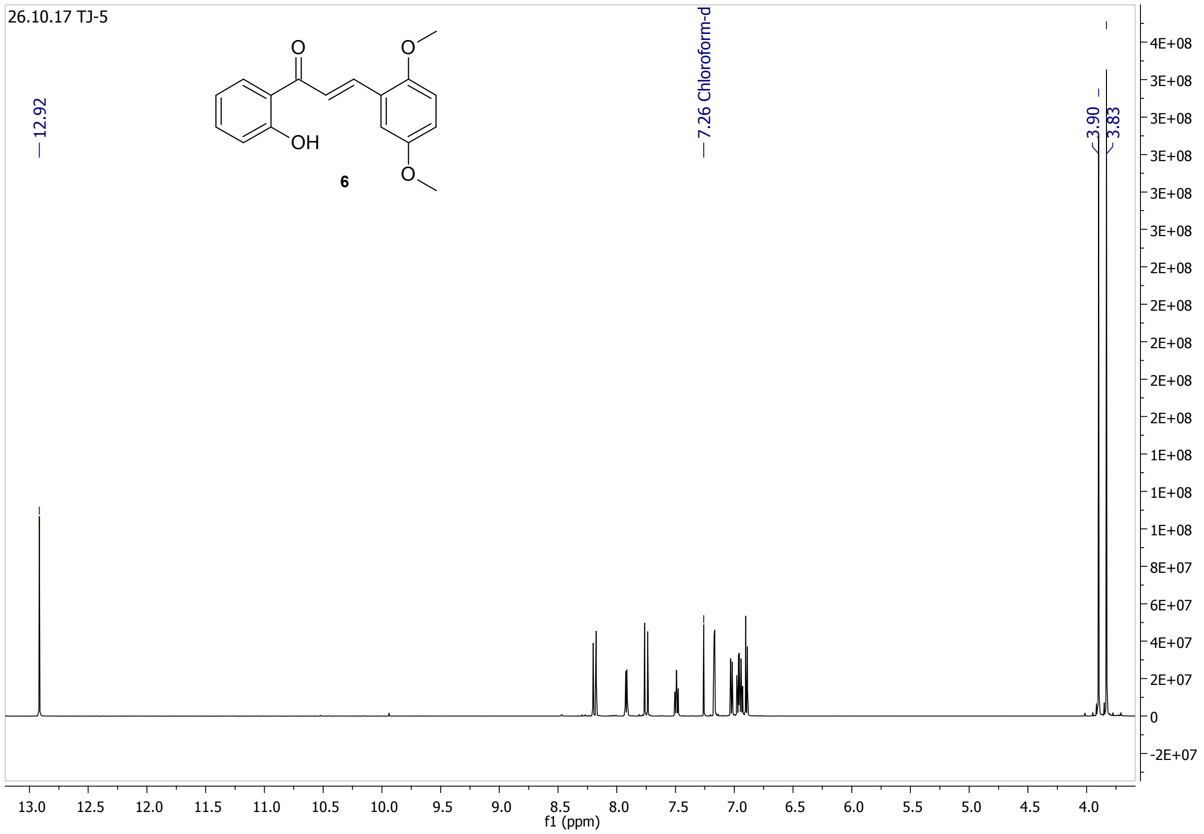

Figure S25. ^1H NMR spectral of 2′-hydroxy-2″,5″-dimethoxychalcone (**6**) ($CDCl_3$, 600 MHz).

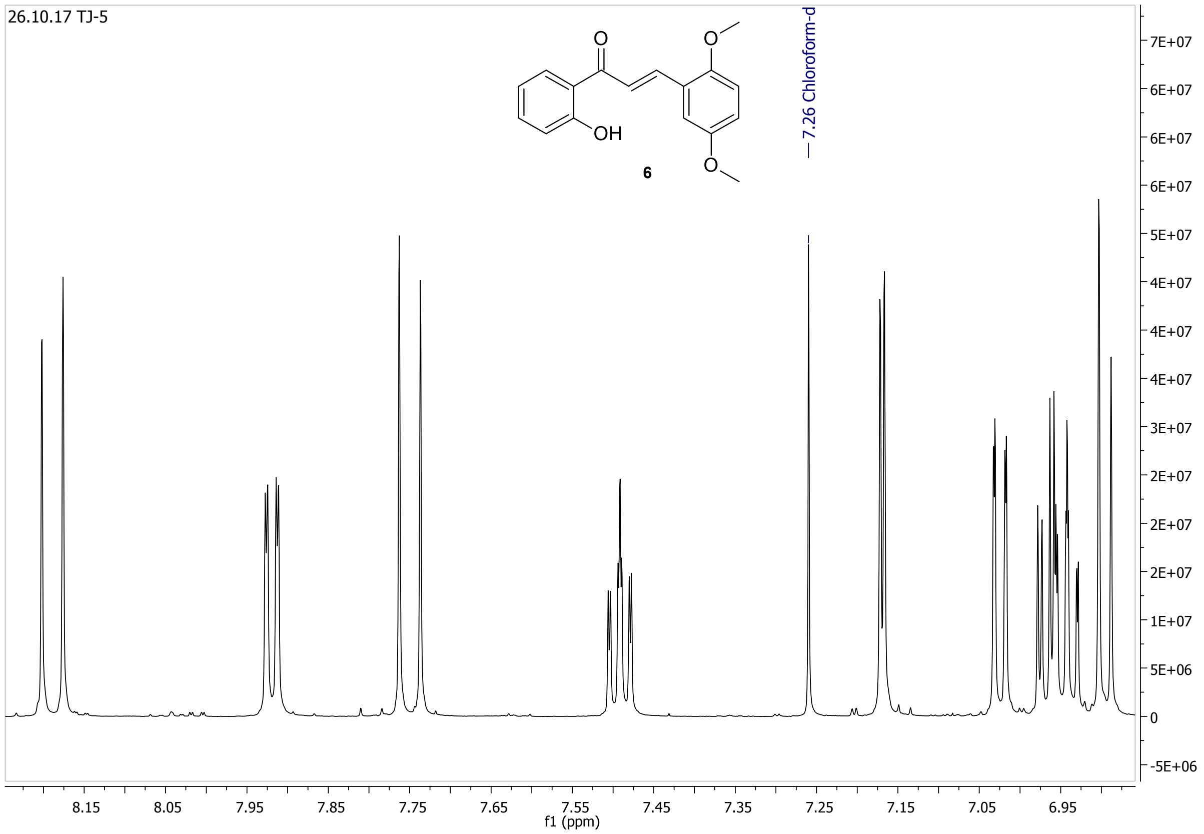

Figure S26. Part of the ^1H NMR spectral 2′-hydroxy-2″,5″-dimethoxychalcone (**6**) ($CDCl_3$, 600 MHz).

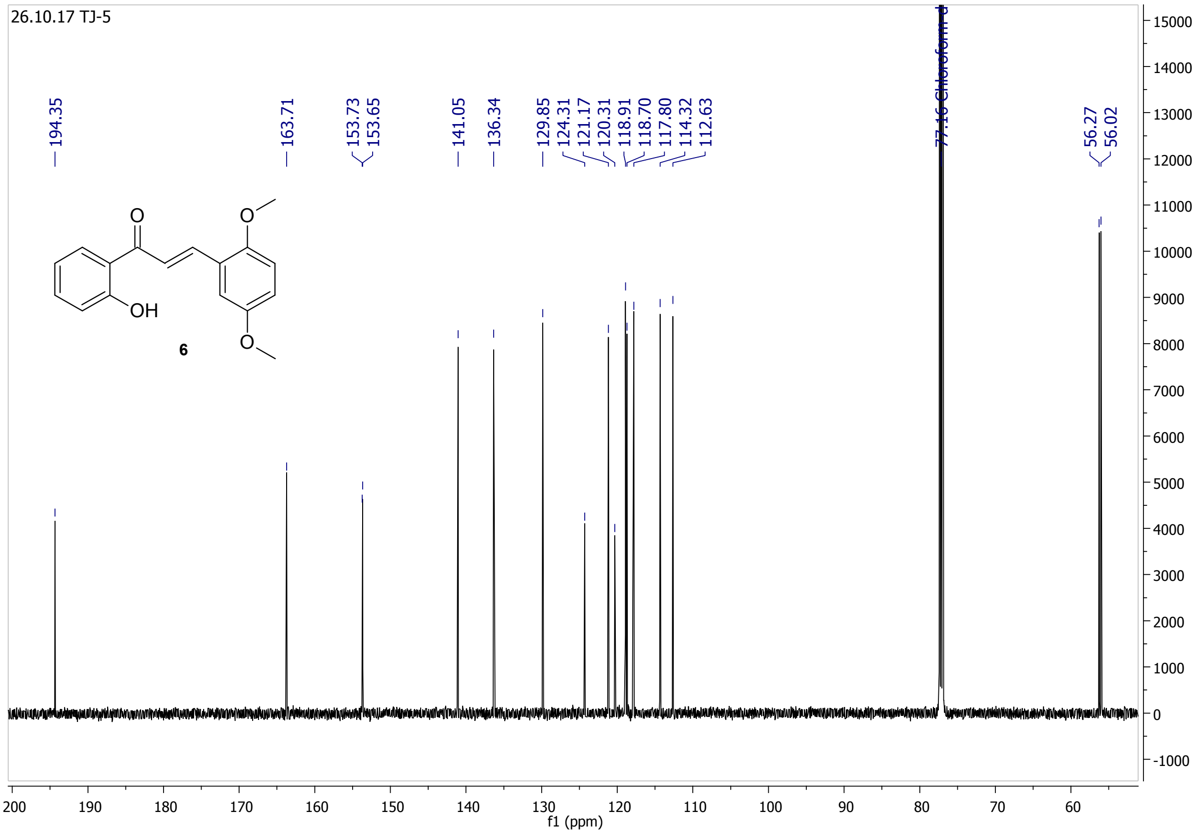

Figure S27. ^{13}C NMR spectral of 2′-hydroxy-2″,5″-dimethoxychalcone (**6**) ($CDCl_3$, 151 MHz).

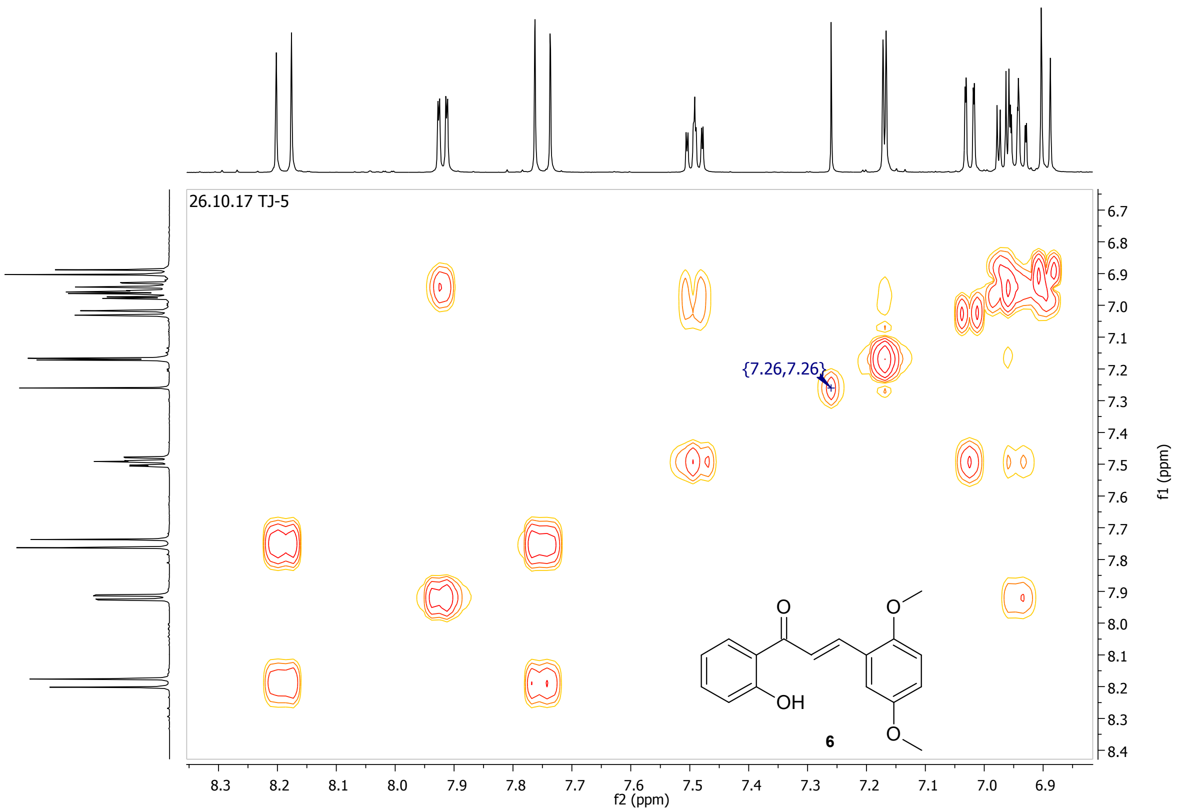

Figure S28. COSY spectral of 2′-hydroxy-2″,5″-dimethoxychalcone (**6**) ($CDCl_3$, 151 MHz).

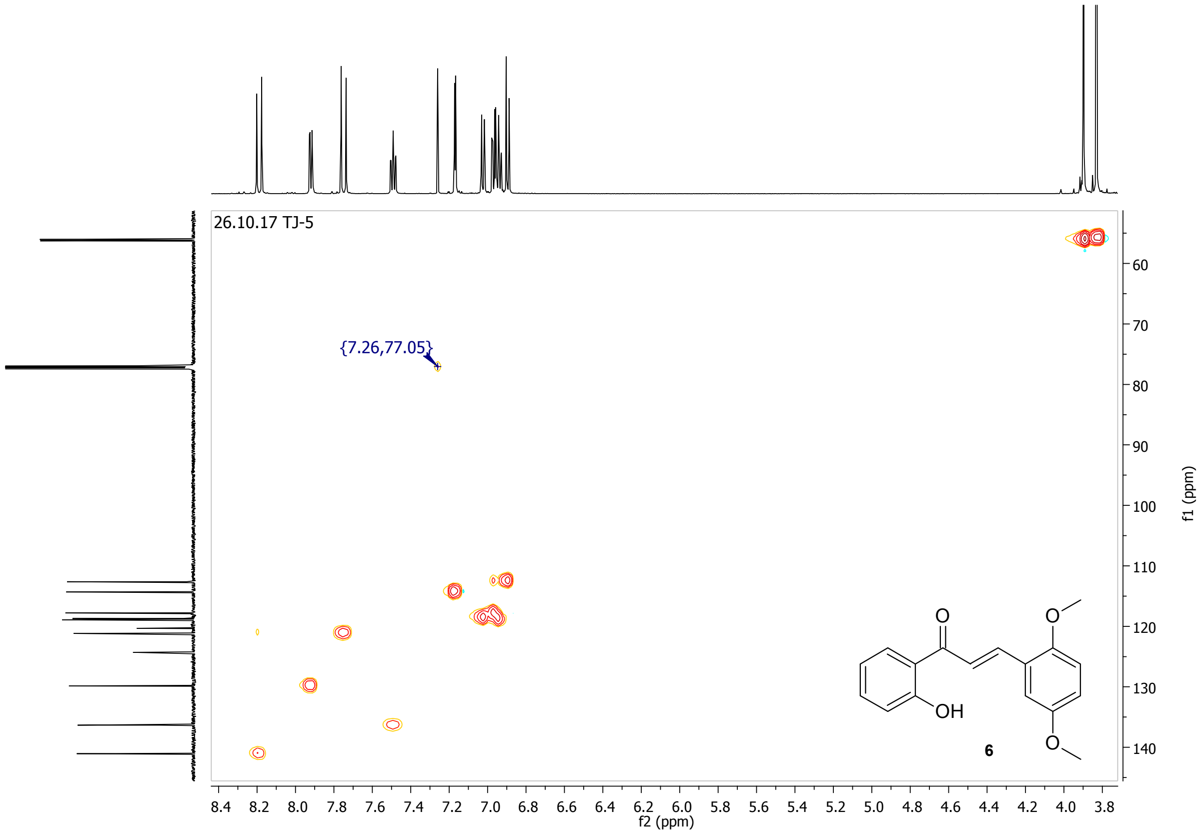

Figure S29. HSQC spectral of 2′-hydroxy-2″,5″-dimethoxychalcone (**6**) ($CDCl_3$, 151 MHz).
